# Molecular classification and prognosis study of pancreatic ductal adenocarcinoma through multi-omics integrated clustering analysis

**DOI:** 10.7717/peerj.20619

**Published:** 2026-02-16

**Authors:** Guodong Zhong, Lei Wang, Peiling Fu, Beibei Xi, Houqiang Li, Yanhong Cheng, Jianlong Lin, Linying Chen

**Affiliations:** 1 The Second Affiliated Hospital of Fujian Traditional Chinese Medical University, Fuzhou, China; 2 The First Affiliated Hospital of Fujian Medical University, Fuzhou, China; 3 Fujian Medical University Union Hospital, Fuzhou, China; 4 Key Laboratory of Cardio-Thoracic Surgery (Fujian Medical University), Fujian Province University, Fuzhou, China; 5 National Regional Medical Center, Binhai Campus of the First Affiliated Hospital of Fujian Medical University, Fuzhou, China; 6 Fujian Provincial Hospital, Fuzhou, China; 7 Fuzhou University Affiliated Provincial Hospital, Fuzhou, China; 8 Shengli Clinical Medical College of Fujian Medical University, Fuzhou, China

**Keywords:** Pancreatic ductal adenocarcinoma, Integrated clustering of multi-omics data, Prognostic model, IL20RB

## Abstract

**Background:**

Pancreatic ductal adenocarcinoma (PDAC) is a lethal malignancy characterized by significant heterogeneity. We conducted a multi-omics integrated clustering analysis to categorize PDAC molecular subtypes.

**Methods:**

Multi-omics data from The Cancer Genome Atlas-Pancreatic Adenocarcinoma (TCGA-PAAD) were integrated using ten clustering algorithms. Comparisons across PDAC subtypes were performed regarding prognosis, gene mutations, pathways, tumor microenvironment (TME), and chemotherapy sensitivity. A prognostic model was constructed utilizing Cox and Lasso regression based on subtype-related genes.

**Results:**

Samples from the TCGA-PAAD cohort were classified into two subtypes. The CS1 subtype was identified as a high-risk, immunosilent subtype, while the CS2 subtype was characterized as a low-risk, immunoactive subtype. Compared to CS2 subtype, CS1 subtype exhibited shorter survival, higher frequency of genetic mutations, more aggressive tumor-promoting nature, lower TME immune score, and increased sensitivity to chemotherapy. The prognostic model related to PDAC subtypes displayed robust predictive efficiency; IL20RB gene emerged having superior predictive capability.

**Conclusions:**

We successfully identified two distinct PDAC subtypes. The developed prognostic model exhibited strong predictive efficacy; and the upregulation of IL20RB was identified as a promising therapeutic target for PDAC.

## Introduction

Pancreatic ductal adenocarcinoma (PDAC) is a highly lethal cancer of the digestive system, characterized by an increasing incidence and high cancer-associated mortality rates (Siegel, Giaquinto & Jemal, 2024). The substantial mortality rate associated with PDAC can be attributed in part to its typically late diagnosis at advanced stages (Ju et al., 2024), aggressive tumor biology, and the lack of effective biomarkers for disease stratification during clinical management. Generally, most cases of PDAC exhibit significant resistance to conventional cytotoxic chemotherapy. Furthermore, the efficacy of emerging therapeutic strategies, including personalized targeting of key oncogenic pathways and immunotherapies targeting the tumor microenvironment (TME), remains limited for most PDAC patients (Ju et al., 2024; Bear, Vonderheide & O’Hara, 2020). Recent research has indicated that subtypes and precursors of PDAC are associated with distinct genetic alterations, histomorphological features, and clinical presentations (Bazzichetto et al., 2020). However, the complex and heterogeneous nature of PDAC, along with its immunosuppressive TME and their interrelations, remains poorly understood. This challenging situation underscores the urgent need for exploration into its biological mechanisms in order to identify new therapeutic targets and improve patient prognosis.

Several studies have elucidated the molecular subtypes of PDAC based on genomic and transcriptomic characteristics (Bazzichetto et al., 2020; Moffitt et al., 2015; Bailey et al., 2016; Hwang et al., 2022). Moffitt et al. (2015) identified two primary subtypes: basal-like and classical, with the former associated with a poorer prognosis. Additionally, Bailey delineated four distinct molecular subtypes of PDAC—pancreatic progenitor, squamous, aberrantly differentiated endocrine exocrine, and immunogenic—each characterized by unique gene mutations and functional attributes. Notably, the squamous subtype exhibited the most unfavorable prognosis, while the immunogenic subtype was linked to immune cell functionality (Bailey et al., 2016). Furthermore, Hwang et al. (2022) classified PDAC into three subtypes—classical, squamous-basal-like, and treatment-enriched—utilizing single-cell RNA sequencing alongside spatial transcriptome analysis. The identification of these molecular subtypes has not only enhanced our understanding of PDAC pathogenesis but also provided valuable insights for developing novel therapeutic strategies aimed at improving treatment efficacy and patient outcomes. However, it is important to note that malignant transformation and tumor development are influenced not solely by transcriptomics but also encompass genomics, epigenetics, proteomics, and metabolomics. Multi-omics studies offer comprehensive molecular information across various levels, facilitating a more systematic understanding of tumor characteristics and progression (Subramanian et al., 2020). The analysis of multi-omics data holds greater significance compared to single-omics approaches (Chakraborty et al., 2018; Rajczewski, Jagtap & Griffin, 2022; Heo et al., 2021).

Currently, research on the multi-omics classification of PDAC remains limited. In response to this gap, we aimed to subtype PDAC utilizing a comprehensive analysis of multi-omics data, which includes mRNA, long non-coding RNA (lncRNA), miRNA, and DNA methylation derived from The Cancer Genome Atlas-Pancreatic Adenocarcinoma (TCGA-PAAD) dataset. We employed a clustering analysis that integrated various algorithms to elucidate the heterogeneity of PDAC and enhance the accuracy of recurrence risk prediction as well as clinical prognosis assessment for this malignancy. Furthermore, we developed a prognostic model for PDAC based on genes associated with specific subtypes. Through this process, we identified key genes most closely linked to patient prognosis and validated these findings experimentally using clinical tissue samples.

## Materials and Methods

### Collection and processing of public data

The “TCGAbiolinks” R package was employed to obtain the training cohort designated as the TCGA-PAAD dataset, which encompasses mRNA, lncRNA, and miRNA data (Colaprico et al., 2016). The UCSC Xena database (https://xenabrowser.net/datapages/) was utilized to download DNA methylation data, gene copy number alteration (CNA) data, and clinical information pertaining to patients in the TCGA-PAAD cohort. Additionally, the “TCGAmutations” R package facilitated the acquisition of gene mutation data for TCGA-PAAD. Cases involving non-pancreatic adenocarcinoma, patients with incomplete overall survival (OS) information or OS durations of less than 10 days, and those lacking gene mutation data were excluded from analysis. Ultimately, a total of 158 PAAD patients were included in this study as the TCGA-PAAD cohort for multi-omics integrated clustering analysis and as the training cohort for prognostic model construction. Furthermore, the GSE183795 dataset, which consists of 139 cases of PDAC, was obtained from the Gene Expression Omnibus (GEO) database (Yang et al., 2022) to serve as an independent validation cohort. Batch effects present in both datasets were mitigated using the “sva” R package (Leek et al., 2012).

### Integration analysis and visualization of multi-omics data

The multi-omics data, encompassing mRNA, lncRNA, miRNA, and DNA methylation, was analyzed for PDAC molecular classification utilizing the “MOVICS” R package (Lu et al., 2021). Initially, prognosis-related molecules were identified from the multi-omics dataset through Cox regression analysis. The optimal number of clusters (k-value) was determined using clustering prediction index (CPI) and gap statistics analysis. Subsequently, ten clustering algorithms—iClusterBayes, moCluster, CIMLR, IntNMF, ConsensusClustering, COCA, NEMO, PINSPlus, SNF, and LRA—were employed to characterize the molecular features of PDAC. Silhouette scores were utilized to quantify sample similarity within each subtype. Finally, the visualization of multi-omics data was accomplished through the use of a complex heatmap.

### Clinical and genetic characteristics of molecular subtypes of PDAC

Next, we analyzed the clinical and genetic characteristics of different PDAC subtypes. Kaplan–Meier survival curves were generated, and the log-rank test was conducted to evaluate the prognostic features among the molecular subtypes of PDAC. The top 20 genes exhibiting the highest mutation frequencies from the TCGA-PAAD cohort were selected, and differences in mutated genes between the two PDAC subtypes were analyzed. Results related to differentially mutated genes were presented in a waterfall plot. The “MOVICS” R package was utilized to analyze fragment genome alteration (FGA), fraction genome loss (FGL), fraction genome gain (FGG), and tumor mutation burden (TMB) across various PDAC subtypes.

### Biological functional analysis of differential expression genes in PDAC

Biological functional analysis was conducted on PDAC molecular subtypes. In brief, the DESeq2 or limma R packages was utilized to perform differential expression genes (DEGs) analysis. DEGs were identified using a threshold of an adjusted *p*-value less than 0.01 and —log2 fold change— greater than 1. The top 100 subtype-related genes for each PDAC subtype were identified, followed by a functional enrichment analysis conducted using the clusterProfiler and GSVA R packages through single sample gene set enrichment analysis (ssGSEA) and gene set variation analysis (GSVA). Additionally, gene ontology (GO) and Kyoto Encyclopedia of Genes and Genomes (KEGG) analyses were carried out. An adjusted *p*-value of less than 0.05 was considered statistically significant as an indicator.

### Evaluation of the TME in PDAC

The IOBR R package was employed for TME analysis, incorporating several algorithms, including ESTIMATE for calculating stromal and immune scores in malignant tumor tissues, CIBERSORT algorithm for assessing immune cell composition, and MCPcounter for quantifying the abundance of fibroblasts and vascular endothelial cells (Zeng et al., 2021). Furthermore, the enrichment of functional pathways associated with immune-related genes, as well as the expression levels of immune checkpoint (IC)-related genes, were compared between the two groups.

### Validation of nearest template prediction and analysis of chemotherapy sensitivity

The nearest template prediction (NTP) is a method utilized for subtype prediction of target samples based on specific gene markers. The GSE183795 cohort was employed to validate the robustness of subtype classification achieved through NTP (Hoshida, 2010). Additionally, the “MOVICS” R package was utilized to assess the chemotherapy sensitivity of two cohorts—the TCGA-PAAD cohort and the GSE183795 cohort—toward four commonly used chemotherapy agents: docetaxel, gemcitabine, paclitaxel, and vinorelbine. This assessment was conducted based on the genomic characteristics of the patients involved.

### Development and validation of a prognostic model associated with molecular subtypes for PDAC

The survival-related genes that exhibited statistically significant associations with outcomes, selected from the top 200 subtype-related genes, were further identified through univariate Cox regression analysis. Subsequently, least absolute shrinkage and selection operator (Lasso) regression analysis was conducted to select model-constructing genes and develop a prognostic model based on the following formula:

\begin{eqnarray*}Risk~score=\sum _{k=1}^{n}coef(genek)\ast expr(genek) \end{eqnarray*}

Herein, “coef (genek)” denoted the coefficient associated with survival-related genes, whereas “expr (genek)” signified the expression level of these genes.

Patients from the TCGA-PAAD cohort were stratified into high-risk and low-risk groups based on the median risk score, which served as the cut-off value. The GSE183795 cohort, along with an integrated dataset comprising both TCGA-PAAD and GSE183795 cohorts, was utilized as the validation set. The predictive sensitivity of the risk score was assessed using the timeROC R package for estimation purposes. Furthermore, principal component analysis (PCA) was conducted utilizing the PCA package to generate corresponding PCA plots. A nomogram was constructed to predict 1-, 2-, and 3-year survival rates in PDAC patients, with its accuracy evaluated through the Hosmer-Lemeshow test.

The receiver operation curve (ROC) curve analysis was employed to evaluate the predictive efficiency of marker genes involved in the development of the prognostic model for clinical outcomes in PDAC. The gene exhibiting the highest predictive efficacy was identified as a potential molecular marker for PDAC, which will be further validated through experiments utilizing human tissue samples. Furthermore, we examined the distribution of marker gene across various PDAC cell types using the tumor immune single-cell hub 2 (TISCH2) (Sun et al., 2021).

### PDAC patients and specimens

A total of 68 consecutive cases from the First Affiliated Hospital of Fujian Medical University were collected between 2020 and 2023. All original Hematoxylin & Eosin (H&E) staining slides were meticulously reviewed, and patients’ clinical pathological parameters were extracted from their medical records. Representative 4-µm sections were prepared on SuperfrostPlus glass slides (Matsunami Glass Industry, Osaka, Japan) for immunohistochemical staining. OS was defined as the duration from the date of initial diagnosis to the date of death. None of the patients had died within one month post-surgery. Additionally, six fresh PDAC specimens along with their adjacent normal tissues were collected from consecutive patients within the cohort, based on tissue availability, and preserved in a −80 °C freezer for subsequent real-time fluorescent quantitative PCR (RT-PCR) and Western blotting (WB) assays. The clinicopathological characteristics of this patient cohort are summarized in Table S4. Written informed consent was obtained from all patients or their legal guardians if applicable. This research project has received approval from the Institutional Research Ethics Committee at the First Affiliated Hospital of Fujian Medical University ([2020]140).

### Experiments of RT-PCR, WB and immunochemistry

The RT-PCR, WB, and immunochemistry (IHC) assays were conducted according to the manufacturers’ specifications. The RT-PCR assay was performed using six pairs of PDAC and adjacent non-tumor tissues. The total mRNA was extracted using the RNeasy Mini Kit (Qiagen, Valencia, CA, USA), followed by reverse transcription with the PrimeScript™ RT Master Mix Kit (Takara, Kyoto, Japan). Quantitative PCR amplification was then performed using TB Green^®^ Premix Ex Taq™ II (Takara). *β*-actin served as the reference gene. The primer sequences for human IL-20RB were as follows: forward primer sequence 5′-AGGCCCAGACATTCGTGAAG-3′, and reverse primer sequence 5′-GCATGAAGCCAACAAAGGCA-3′. All PCR samples were quantified utilizing the comparative CT method to determine relative mRNA levels.

The WB analysis was performed using six pairs of PDAC and adjacent non-tumor tissues. The total proteins were extracted using a phosphoprotein extraction kit (KGI Bio, Shanghai, China) and quantified with a BCA Protein Assay kit (Biyuntian, Shanghai, China). The proteins were then separated and transferred onto polyvinylidene difluoride membranes. The membranes were blocked using a fast blocking solution (Biyuntian) and subsequently incubated overnight at 4 °C with primary antibodies against the interleukin-20 receptor subunit beta (IL-20RB, Solebo, China; K008033P). Following washing steps, the membranes were incubated with HRP-conjugated secondary antibodies (Cell Signaling Technology, Danvers, MA, USA; #7074) and enhanced using a chemiluminescence HRP substrate (Bole ChemiDocTM, America).

The IHC analysis was performed using 68 pairs of PDAC and adjacent non-tumor tissues. The anti-IL20RB antibody (Solebo; K005599P) was applied using the Dako EnVision FLEX+ detection system (Dako, Glostrup, Denmark) along with a DAB detection kit (Ventana, Export, PA, USA). This process followed deparaffinization, hydration, and antigen retrieval in EDTA buffer at pH 8.0 for 30 min at 37 °C. The IL20RB staining results were evaluated by two pathologists. The IL20RB scores were categorized into four grades: 0 = negative, 1 = weak, 2 = moderate, and 3 = strong. These grades were subsequently multiplied by the percentage of positively stained cells observed in the cytoplasm of tumor cells to derive the final IHC scores. Scores equal to or less than 40 were classified as negative results, while scores exceeding 40 were considered positive results.

### Statistical analysis

All statistical analyses were performed using R language (Version 4.2.0; R Core Team, 2022) and GraphPad Prism software (Version 9.5.0). The *t*-test or Wilcoxon test was employed to compare measurement data between two groups, while analysis of variance (ANOVA) or the Kruskal–Wallis test was utilized for comparisons among three or more groups. Correlation coefficients between two continuous variables were calculated using either the Spearman or Pearson methods. The chi-square test or Fisher’s exact test was applied to compare count data across different groups. Kaplan–Meier survival analysis and log-rank tests were used to assess survival differences between the groups. The hazard ratio (HR) for each group was determined through univariate and multivariate Cox regression analyses. A significance level of *P* < 0.05 was considered indicative of a statistically significant difference.

## Results

### Multiomics-related molecular subtypes of PDAC

**Figure 1:**
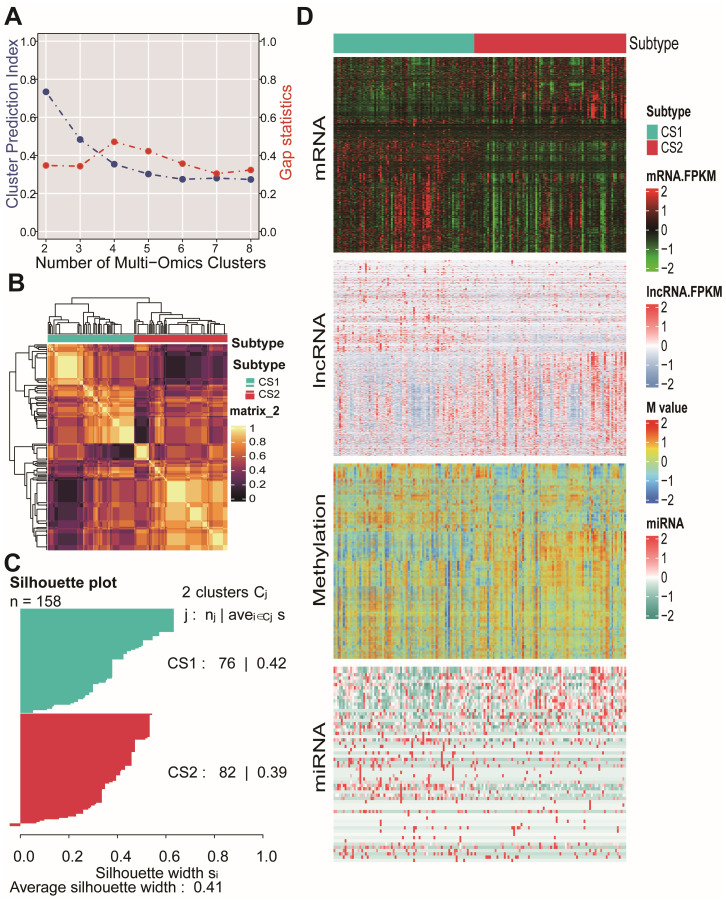
Molecular classification of PDAC based on multi-omics analysis. (A) The clustering prediction index (CPI) was represented by the blue line, while the gap statistics was illustrated by the red line for the TCGA-PAAD cohort. (B) A heatmap illustrated molecular clustering across different molecular subtypes. (C) The silhouette plot quantified sample similarity within each subtype. (D) A complex heatmap displayed molecular markers from each omics layer across different molecular subtypes. PDAC: pancreatic ductal adenocarcinoma; TCGA -PAAD: The Cancer Genome Atlas-Pancreatic Adenocarcinoma.

According to the analysis conducted using ten multiomics ensemble clustering algorithms, the optimal number of clusters for prognosis-related molecular classifications within the TCGA-PAAD cohort was determined to be *k* = 2. This determination was based on both the CPI and gap statistics (Fig. 1A). The two identified molecular subtypes within the TCGA-PAAD cohort were designated as CS1 (*n* = 76) and CS2 (*n* = 82), with their associated molecular characteristics illustrated in Fig. 1B. The similarity of samples within each subtype was reflected in their silhouette scores, which were 0.42 for CS1 and 0.39 for CS2 (Fig. 1C). Subsequently, these subtypes were further integrated through a consensus ensemble approach that highlighted distinct molecular expression patterns including mRNA, long non-coding RNA (lncRNA), microRNA (miRNA)—and DNA methylation profiles (Fig. 1D). The inclusion of multiple RNA types provides a more comprehensive view of the regulatory landscape underlying the PDAC subtypes.

### Clinicopathological characteristics and genetic mutation in different PDAC subtypes

**Figure 2:**
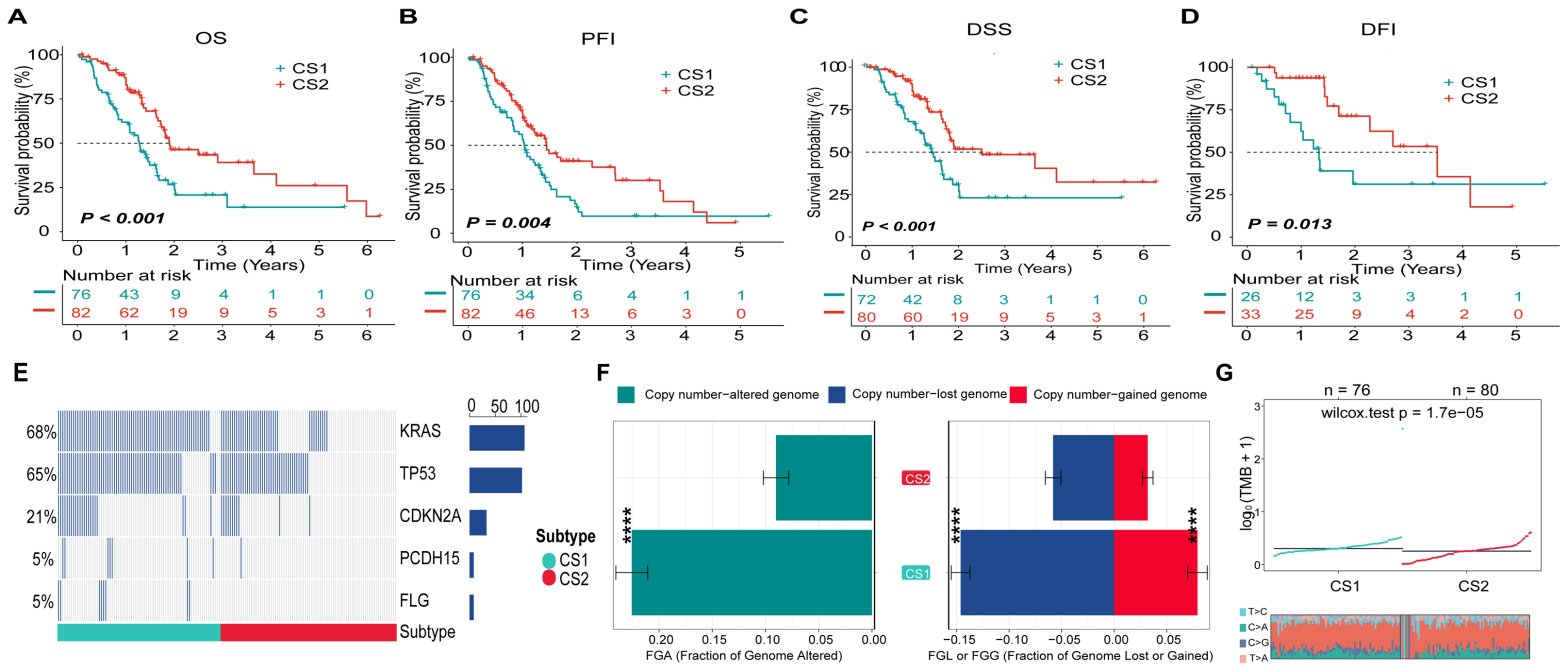
Survival curves of clinical prognoses and analysis of genetic mutations in PDAC subtypes. (A) Overall survival (OS) curve for different PDAC subtypes. (B) Progression-free interval (PFI) curve for various PDAC subtypes. (C) Disease-specific survival (DSS) curve across PDAC subtypes. (D) Disease-free interval (DFI) curve among the distinct PDAC subtypes. (E) The waterfall plot illustrated significant differences in gene mutations across five genes between the PDAC subtypes. (F) Comparison of genomic alterations, specifically FGA and FGG/FGL, between the two identified PDAC subtypes; data were expressed as mean ± standard deviation. (G) Comparative analysis of tumor mutation burden (TMB) between the two distinct PDAC subtypes.

In terms of the relationships between clinicopathological characteristics, the CS1 subtype demonstrated a higher tumor grade (*P* < 0.05, Table S1) and exhibited poorer OS, progression-free interval (PFI), disease-specific survival (DSS), and disease-free interval (DFI) when compared to the CS2 subtype (*P* < 0.001, *P* < 0.01, *P* < 0.001, and *P* < 0.05 respectively; Figs. 2A–2D). In addition, genetic mutation analysis revealed that the most frequently mutated genes—namely KRAS, TP53, CDKN2A, PCDH15, and FLG—showed significantly higher mutation frequencies in the CS1 subtype than in the CS2 subtype (Table S2; Fig. 2E). Furthermore, FGA, FGL, and FGG values for the CS1 subtype were significantly elevated compared to those of the CS2 subtype (*P* < 0.0001; Fig. 2F). Additionally, the mean TMB value for the CS1 subtype was recorded at 0.30, which was significantly greater than that observed in the CS2 subtype (*P* < 0.001; Fig. 2G).

### Differential gene enrichments and TME in PDAC subtypes

**Figure 3:**
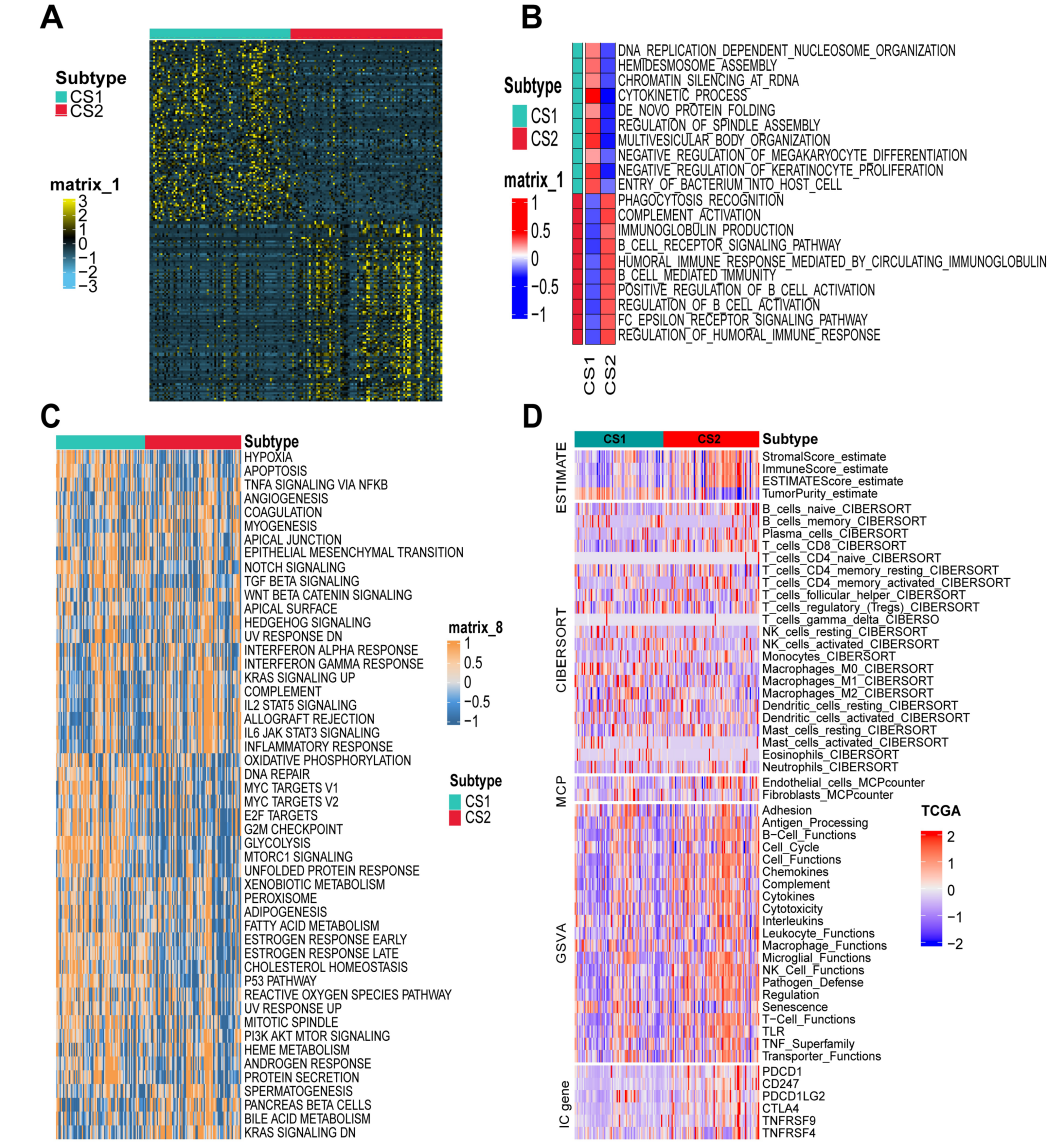
Pathway enrichment and tumor microenvironment (TME) analysis in different PDAC subtypes. (A) The heatmap illustrated the top 100 subtype-related genes that were specifically upregulated in each PDAC subtype. (B) The single-sample gene set enrichment analysis (ssGSEA) was performed for each subtype within the TCGA-PAAD cohort, with pathways represented in red indicating higher enrichment scores. (C) The heatmap presented the results of the Hallmark gene set variation analysis across each subtype. (D) This heatmap depicted the immune stromal score, distributions of immune cells, endothelial cells, and fibroblasts, as well as immune pathway enrichment and the distribution of immune checkpoint-related genes across two PDAC subtypes from the TCGA-PAAD cohort. ESTIMATE, estimation of stromal and immune cells in malignant tumor tissues using expression data; GSVA, gene set variation analysis; MCP, McCulloch-Pitts.

To investigate the differences between the two clustering results, we identified the top 100 genes that were specifically upregulated in each subtype (Fig. 3A, Table S3). Following this, pathway enrichment analysis was conducted using both ssGSEA (Fig. 3B) and GSVA (Fig. 3C) algorithms. The CS1 subtype exhibited significant enrichment in pathways such as the p53 signaling pathway, cell cycle regulation pathways, DNA damage repair mechanisms, checkpoints of cell cycle regulation from G2 to mitosis phase, gene expression regulated by E2F transcription factors, G1/S-specific transcription processes, Myc target pathways, TGF-*β* signaling pathways, and TNF-*α*-NF*κ*B pathways. In contrast, the CS2 subtype demonstrated predominant enrichments in inflammation-related processes including the IL-6/JAK/STAT3 signaling pathway and xenograft rejection mechanisms.

Subsequently, we conducted a more in-depth analysis of the immune microenvironments associated with the CS1 and CS2 phenotypes. As anticipated, the CS2 subtype exhibited significantly elevated immunity scores, stromal scores, ESTIMATE scores, and levels of immune cells within the TME when compared to those observed in the CS1 subtype. Notably, there was also a marked increase in quantities of CD8+ T cells, B cells, plasma cells, NK cells, monocytes, activated mast cells, and neutrophils in the CS2 subtype. Additionally, IC-related genes such as PDCD1, CD247, CTLA4, TNFRSF9, and TNFRSF4 were also found to be significantly upregulated in the CS2 subtype (Fig. 3D).

### NTP classification and validation using an external cohort

**Figure 4:**
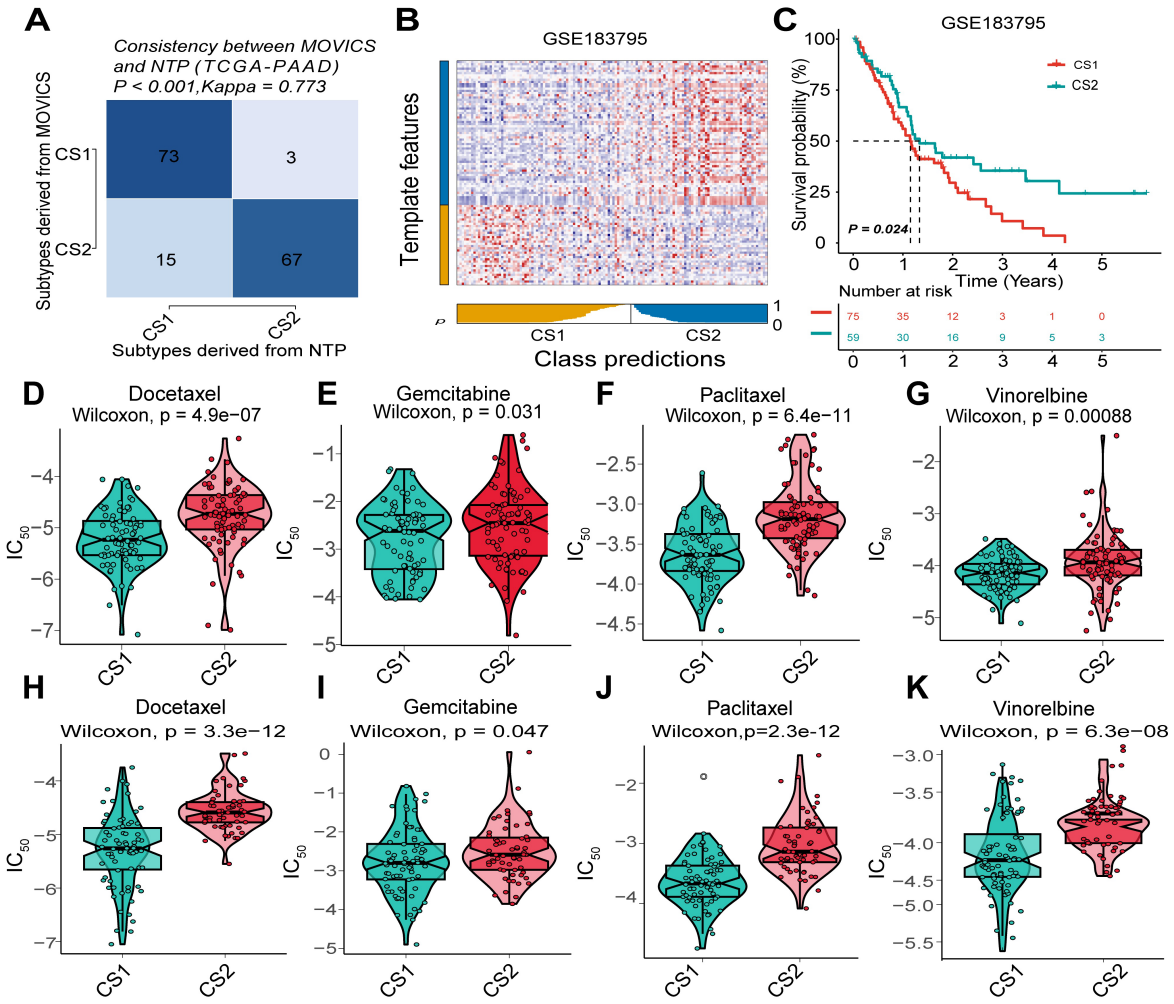
External validation of PDAC multi-omics clustering classification and assessment of chemotherapy sensitivity across two subtypes. (A) Consistency analysis comparing PDAC classifications derived from the NTP classification and multi-omics clustering in the TCGA-PAAD cohort. (B) A heatmap illustrated the distribution of subtype-related genes for CS1 and CS2 subtypes as predicted by the NTP algorithm in the GSE183795 cohort. (C) Kaplan–Meier survival curves along with log-rank tests demonstrated overall survival in the two PDAC subtypes within the GSE183795 cohort. (D–G) Comparative analysis of IC50 values for docetaxel, gemcitabine, paclitaxel, and vinorelbine between both subtypes in the TCGA-PAAD cohort. (H–K) Comparative analysis of IC50 values for docetaxel, gemcitabine, paclitaxel, and vinorelbine between both subtypes in the GSE183795 cohort. NTP, Nearest template prediction; IC_50_, half maximal inhibitory concentration.

Based on the results of differential expression analysis through multi-omics clustering classification, we employed the NTP algorithm to assess the stability of molecular subtypes. The Kappa test revealed a high degree of consistency in predicting the molecular subtypes of PDAC between the two classifications (Kappa value = 0.773, *P* < 0.001; Fig. 4A). The heatmap illustrated the distributions of subtype-related genes for CS1 and CS2 subtypes within the GSE183795 cohort as predicted by the NTP algorithm (Fig. 4B). In alignment with the multi-omics clustering classification, patients classified under the CS2 subtype within the NTP classification demonstrated longer survival times compared to those with the CS1 subtype in the GSE183795 cohort (*P* < 0.05; Fig. 4C).

### Assessment of chemotherapy sensitivity in different PDAC subtypes

The half maximal inhibitory concentration (IC50) values for docetaxel, gemcitabine, paclitaxel, and vinorelbine were observed to be significantly lower in patients with the CS1 subtype compared to those with the CS2 subtype (all *P* < 0.01) within both the TCGA-PAAD cohort (Figs. 4D–4G) and the GSE183795 cohort (Figs. 4H–4K). Consequently, it was suggested that patients with the CS1 subtype may potentially derive greater therapeutic benefits from these chemotherapeutic agents than their counterparts with the CS2 subtype.

### Development of a prognostic model associated with molecular subtypes for PDAC

**Figure 5:**
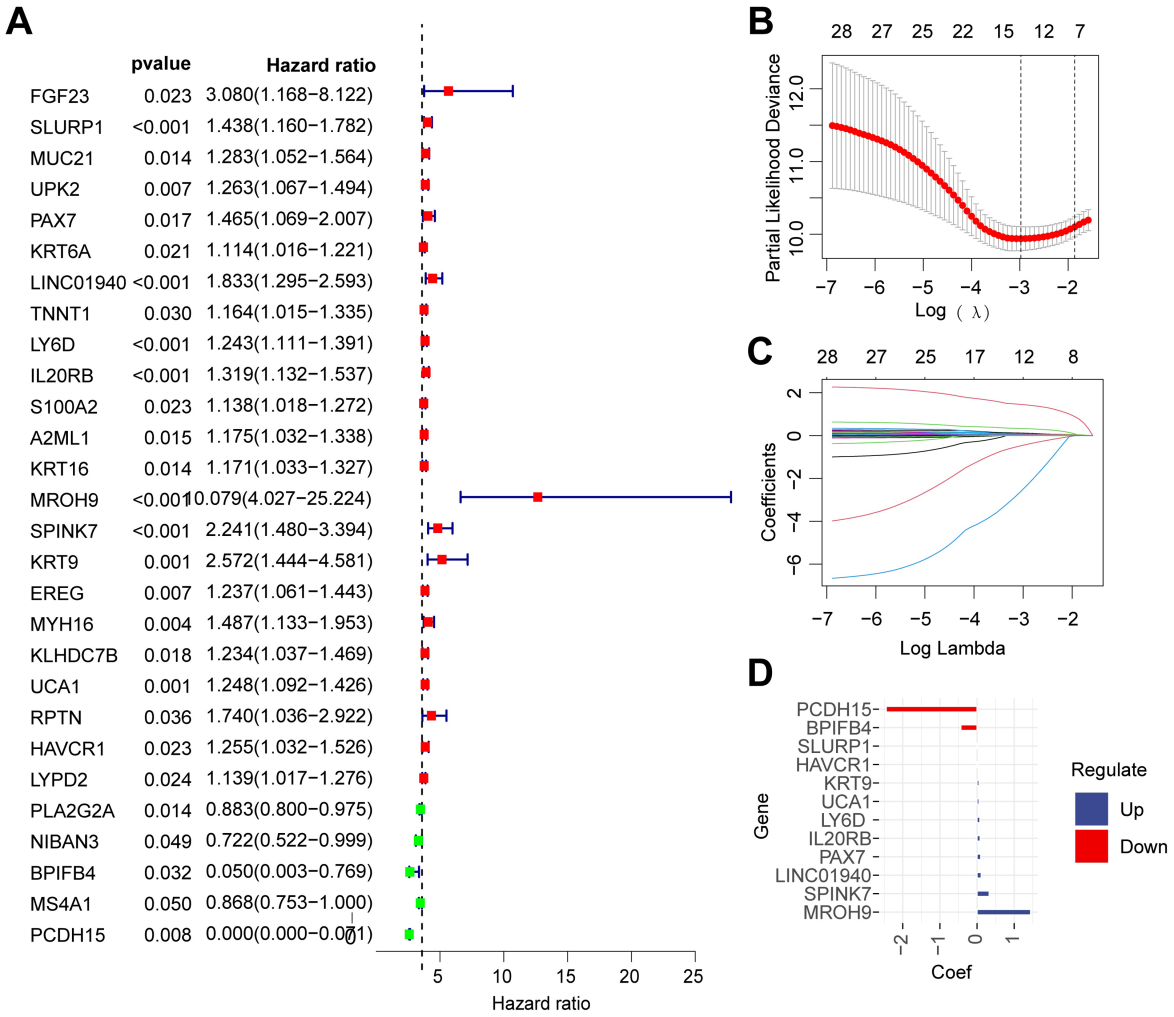
Construction of a prognostic model for PDAC. (A) The forest plot illustrated the selection of 28 survival-related genes derived from subtype- related genes through univariate Cox regression analysis. (B) The parameter plot demonstrated optimal model fitting, indicated by a log *λ* value of −3 in Lasso regression analysis. (C) The coefficient distribution diagram presented the coefficients of each survival-related gene at varying log *λ* values, with each curve representing a distinct survival-related gene. (D) The bar chart displayed the 12 selected genes involved in model construction along with their corresponding coefficients.

A total of twenty-eight survival-related genes were identified through univariate Cox regression analysis from the top 200 subtype-related genes, which were selected based on the highest absolute log2 fold change from the differential expression analysis between CS1 and CS2 subtypes (Fig. 5A). The results of the Lasso regression analysis demonstrated that the model achieved optimal fit, indicated by a minimum partial likelihood deviation value, when the *λ* value was set to −3 on the log(*λ*) curve (Fig. 5B). Consequently, a total of 12 genes (SLURP1, PAX7, LINC01940, LY6D, IL20RB, MROH9, SPINK7, KRT9, UCA1, HAVCR1, BPIFB4 and PCDH15) were identified as optimal candidate genes for the construction of a prognostic model (Fig. 5C). The TCGA-PAAD cohort served as the training dataset; risk scores for each sample were calculated based on the correlation coefficients and expression levels of the model-constructing genes (Fig. 5D).

**Figure 6:**
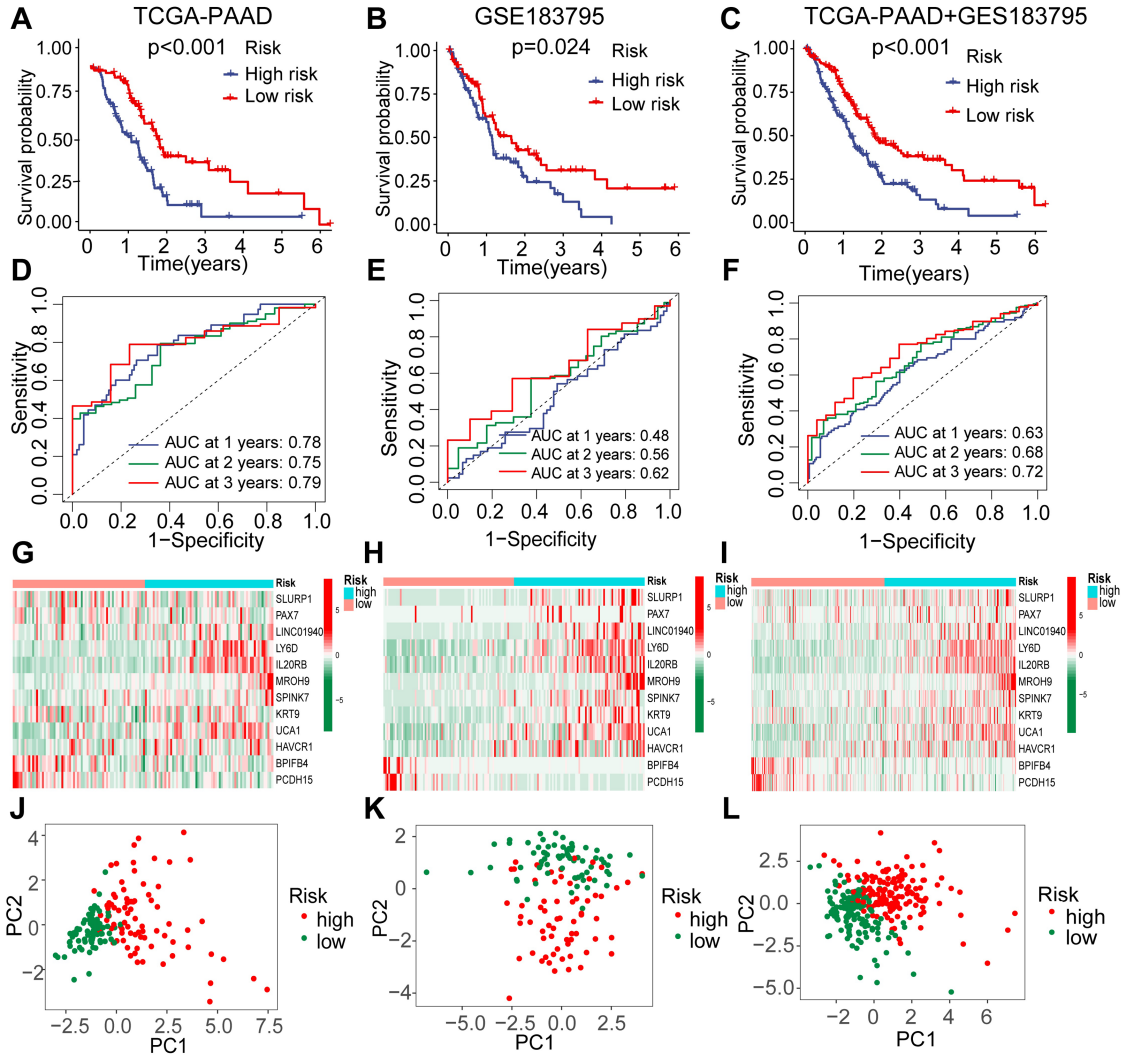
Visualization analysis and efficacy validation of the PDAC prognostic model. (A–C) The overall survival rates for high-risk and low-risk groups of PDAC patients were analyzed using Kaplan–Meier and log-rank methods across the training cohort, validation cohort, and integration cohort, respectively. (D–F) Receiver operating characteristic (ROC) curves for 1-year, 2-year, and 3-year risk scores derived from the prognostic model were generated in the training cohort, validation cohort, and integration cohort, respectively. (G–I) Heatmaps illustrated significant differences in the expression levels of twelve genes used to construct the model between high-risk and low-risk groups within the training cohort, validation cohort, and integration cohort. (J–L) Principal component analysis (PCA) plots demonstrated that the model effectively classified samples into high-risk and low-risk categories across the training cohort, validation cohort, and integration cohort. ROC, receiver operating curve; PCA, principal component analysis; PC, principal component.

Afterward, we categorized PDAC into high-risk and low-risk groups using a median risk score of −0.367 as the threshold in the TCGA-PAAD training cohort. The risk score was calculated for each patient based on the formula described in the Methods (‘Development and validation of a prognostic model associated with molecular subtypes for PDAC’). We found that patients classified as high-risk exhibited significantly poorer outcomes (*P* < 0.01, Fig. 6A). The area under the curve (AUC) values for 1-year, 2-year, and 3-year survival were recorded at 0.78, 0.75, and 0.79, respectively (Fig. 6D). To confirm the reliability and stability of our prognostic model, we validated it using additional independent validation datasets. Similarly, PDAC patients in the GSE183795 dataset and in an integrated cohort comprising TCGA and GSE183795 were stratified based on the median risk score; both groups demonstrated significantly worse outcomes for those identified as high-risk (Figs. 6B, 6C). In the GSE183795 dataset, AUC values for survival at years one, two, and three were observed to be 0.48, 0.56, and 0.62 (Fig. 6E), while in the integrated cohort these values were noted to be 0.63, 0.68, and 0.72 for corresponding time points (Fig. 6F). Furthermore, significant differences were observed regarding twelve model-constructing genes between high-risk and low-risk groups (Figs. 6G–6I), with PCA analysis revealing two distinct patient clusters across all cohorts (Figs. 6J–6L).

### Development of a nomogram for the prognostic model associated with risk scores

**Figure 7:**
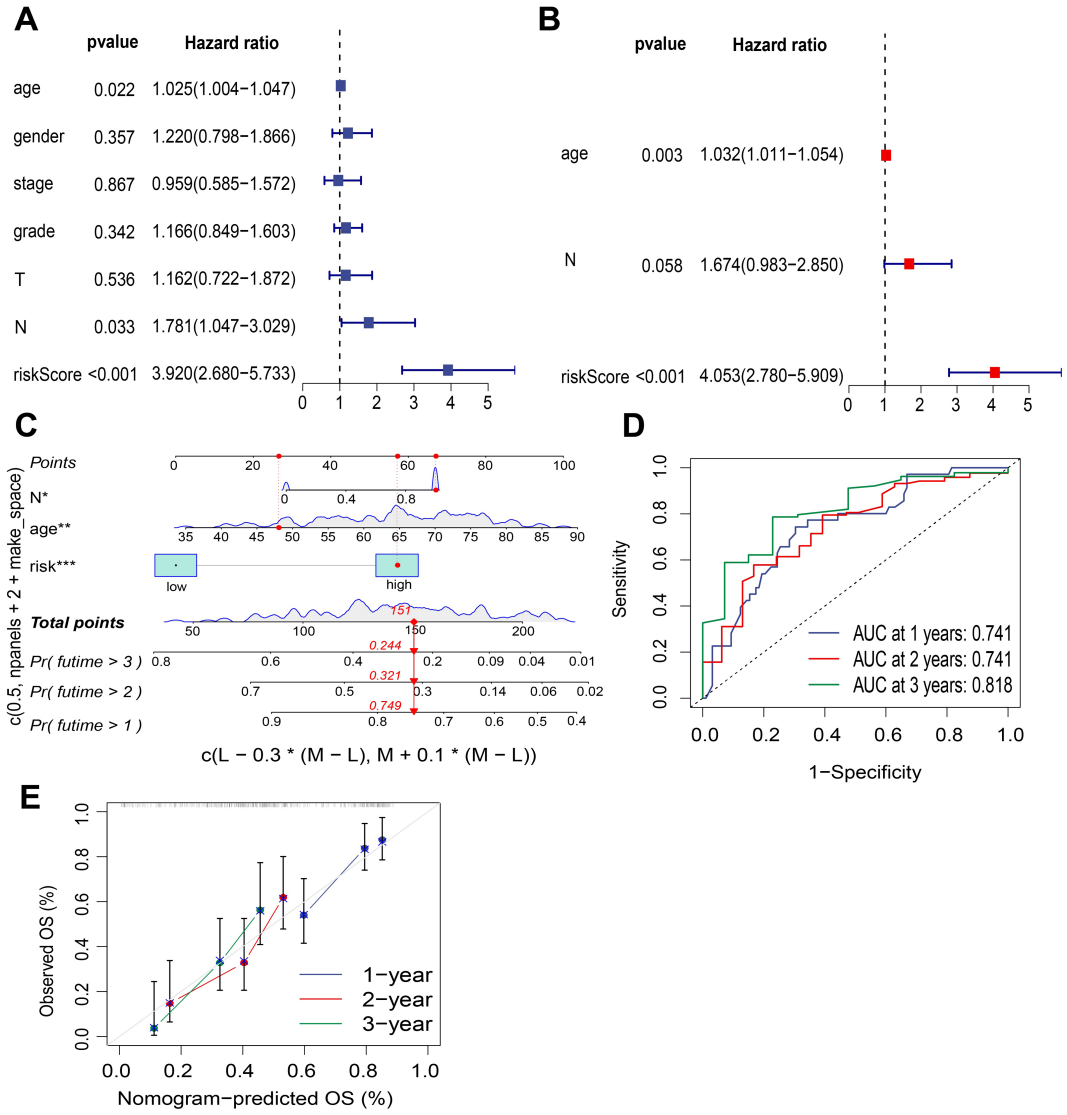
Nomogram construction of the risk score from the prognostic model and clinicopathological characteristics. (A) Univariate Cox regression analysis examining the relationship between the risk score of the prognostic model and various clinicopathological characteristics. (B) Multivariate Cox regression analysis assessed the impact of the risk score from the prognostic model alongside clinicopathological features. (C) The nomogram illustrated predictions for overall survival at 1-year, 2-year, and 3-year intervals based on both risk scores and clinicopathological characteristics. (D) The ROC curves corresponding to 1-year, 2-year, and 3-year outcomes was determined by the nomogram. (E) The calibration curve evaluated the accuracy of predictions made by the nomogram; in this curve, the horizontal axis represented predicted probabilities while the vertical axis denoted actual probabilities. The proximity of the calibration curve to the diagonal dotted line indicated greater predictive accuracy regarding patient survival outcomes. OS, overall survival.

Both univariate and multivariate Cox regression analyses indicated that the patient’s age, pN stage, and risk score were independent prognostic factors in PDAC (for risk score: HR = 4.053, 95% CI [2.780–5.909], *P* < 0.001; Figs. 7A, 7B). Consequently, these variables—age, pN stage, and risk score—were integrated to develop a nomogram for predicting OS at 1-, 2-, and 3-year intervals (Fig. 7C). In this nomogram, the risk score played a significant role in estimating survival probability, serving as both a quantitative and visual tool for forecasting outcomes at the specified time points. The area under the curve (AUC) values for OS at the 1-, 2-, and 3-year marked within the nomogram were recorded as 0.741, 0.741, and 0.818 respectively, indicating strong prognostic capability (Fig. 7D). Additionally, calibration curves were generated to evaluate the performance of the nomograms; The proximity of the calibration curve to the diagonal dotted line indicated that the nomogram effectively predicted patient survival outcomes (Fig. 7E). Furthermore, these findings aligned with results from pathway enrichment analysis and TME analysis conducted during multi-omics classification studies (Fig. S1).

### Levels of gene expression involved in the development of the prognostic model

**Figure 8:**
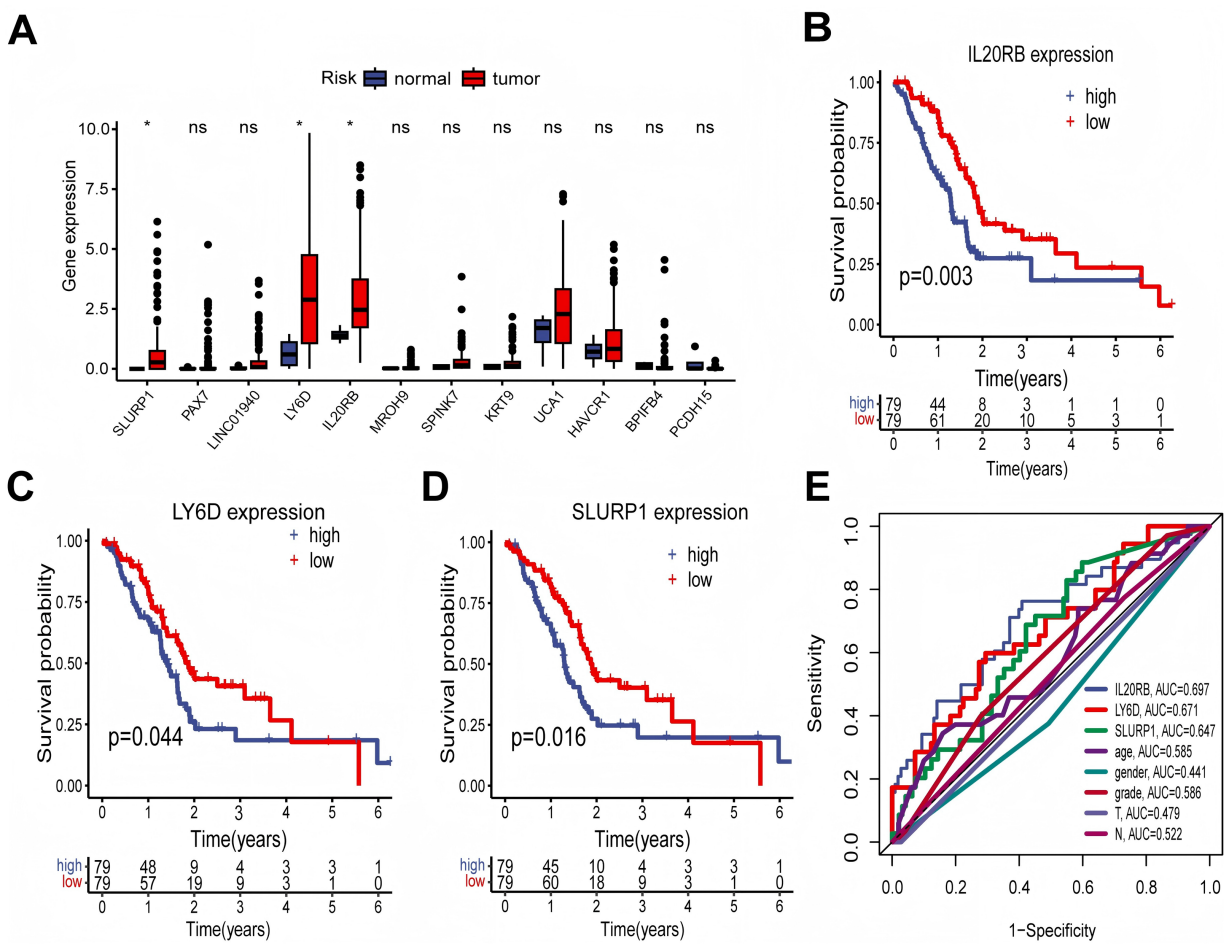
Expression levels of genes involved in the model construction and their association with clinical outcomes. (A) The box plots illustrated the differences in mRNA expression levels of twelve genes implicated in the development of the prognostic model between PDAC tissues and adjacent normal tissues within the TCGA-PAAD cohort. (B–D) The Kaplan–Meier survival analysis, along with log-rank tests, was employed to assess the correlation between IL20RB, LY6D, and SLURP1 gene expression levels and overall survival rates among PDAC patients in the TCGA-PAAD cohort. (E) The ROC curve demonstrated the AUC for predicting prognosis based on clinicopathological characteristics as well as mRNA expression levels of IL20RB, LY6D, and SLURP1 individually. AUC: area under curve.

To comprehensively evaluate the clinical implications of genes with high risk scores, we conducted a screening of 12 model-constructing genes in PDAC patients utilizing the TCGA-PAAD database. In comparison to adjacent normal tissues, only three genes—IL20RB, LY6D, and SLURP1—exhibited significantly elevated mRNA levels in PDAC tissues relative to adjacent normal tissues (*P* < 0.05; Fig. 8A). Notably, all three genes were associated with poor prognostic outcomes in PDAC patients (*P* = 0.003, 0.044, and 0.016 respectively; Figs. 8B–8D). Based on the AUC values for these three genes, we identified IL20RB as a potential prognostic marker for PDAC (Fig. 8E), which was further validated using single-cell RNA sequencing datasets and clinical specimens beyond those available from TCGA data.

**Figure 9:**
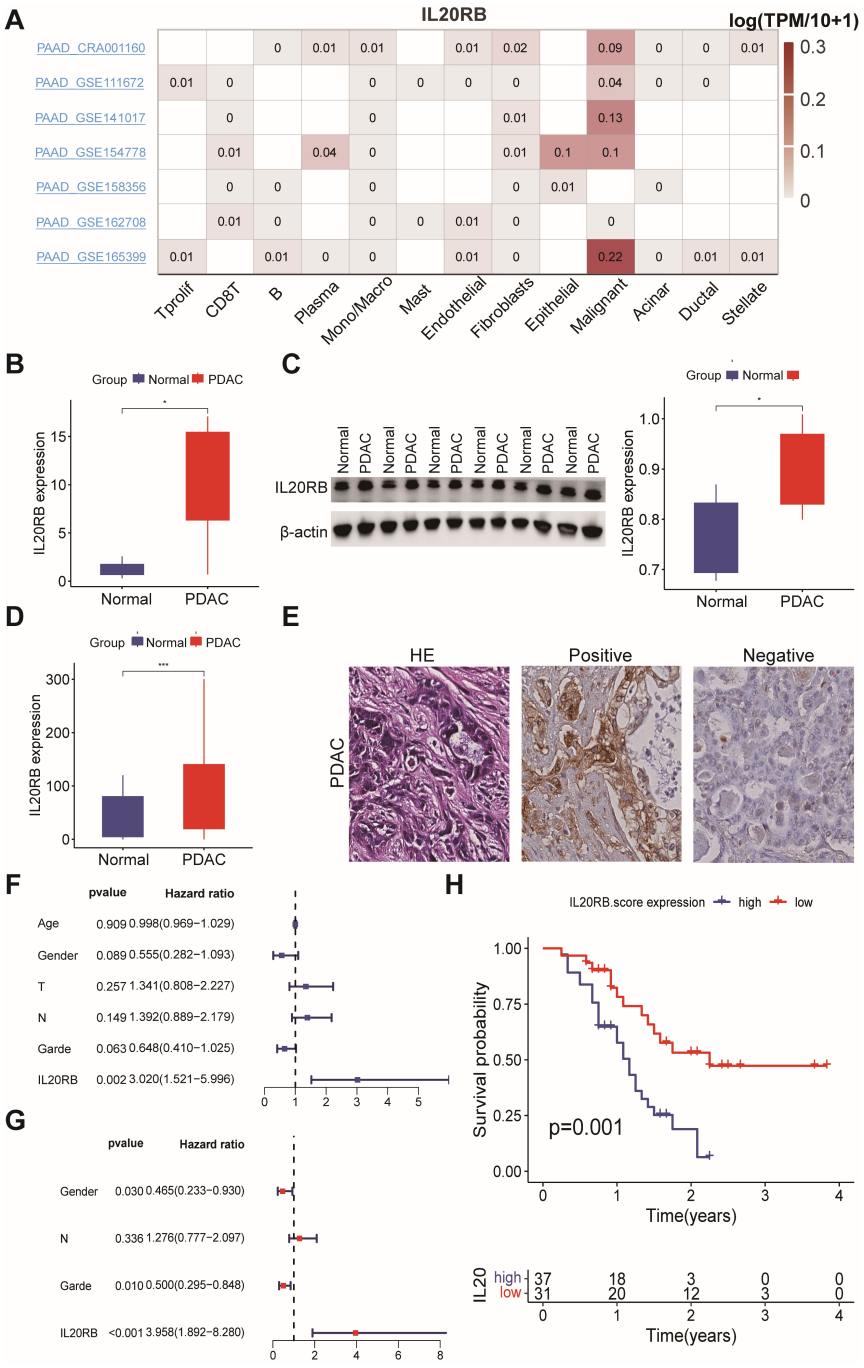
IL20RB levels in PDAC tissues and adjacent normal tissues. (A) The heatmap illustrated the expression distribution of IL20RB across various cell types in five single-cell RNA sequencing datasets of pancreatic cancer. (B) Comparison of mRNA levels of IL20RB between PDAC tissues and adjacent normal tissues was performed using RT-PCR (*P* < 0.05). (C) Protein levels of IL20RB were compared between PDAC tissues and adjacent normal tissues through Western blot analysis (*P* < 0.01). (D) A comparison of protein levels of IL20RB between PDAC tissues and adjacent normal tissues was also conducted *via* immunohistochemistry (*n* = 68, *P* < 0.001). (E) Hematoxylin & Eosin staining, positive and negative expressions of IL20RB protein in IHC analysis were presented (scale bar = 50 mm). (F) Univariate Cox regression analysis was performed to evaluate the impact of variables including the IL20RB protein level determined by IHC alongside clinicopathological characteristics on clinical prognosis. (G) Multivariate Cox regression analysis was subsequently conducted to assess the influence of these variables on clinical prognosis as well. (H) The relationship between overall survival rates in PDAC patients and IL20RB protein levels identified through IHC testing was analyzed using Kaplan–Meier survival curves and log-rank tests.

Firstly, five single-cell RNA sequencing datasets of pancreatic cancer were retrieved to evaluate the mRNA levels of IL20RB across different PDAC cell types using the TISCH2 website. The results indicated that IL20RB was expressed in pancreatic cancer cells in all five datasets, as well as in endothelial cells, fibrocytes, plasma cells, dendritic cells, and monocytes (Fig. 9A). Subsequently, we investigated IL20RB expression in clinical surgical specimens from PDAC patients. The findings demonstrated that IL20RB expression was elevated in tumor tissues compared to their corresponding adjacent normal tissues at both mRNA (Fig. 9B) and protein levels (Fig. 9C). Immunohistochemical staining for IL20RB revealed no to minimal staining in normal pancreatic ductal epithelial cells; conversely, moderate to strong staining was observed in cancerous tissues (Figs. 9D, 9E). Elevated IL20RB expression emerged as an independent adverse prognostic factor for PDAC patients according to Cox regression analysis encompassing various variables such as age, gender, tumor grade, tumor T stage, N stage, and IL20RB expression within the clinical cohort (*n* = 68; hazard ratio = 3.958; 95% CI [1.892–8.280]; *P* < 0.001; Figs. 9F, 9G). Patients exhibiting low levels of IL20RB protein demonstrated significantly longer OS when analyzed using Kaplan–Meier and log-rank methods (Fig. 9H).

### PDAC exhibiting elevated IL20RB expression showed comparable pathway enrichment and chemotherapy sensitivity when compared to the CS1 subtype

**Figure 10:**
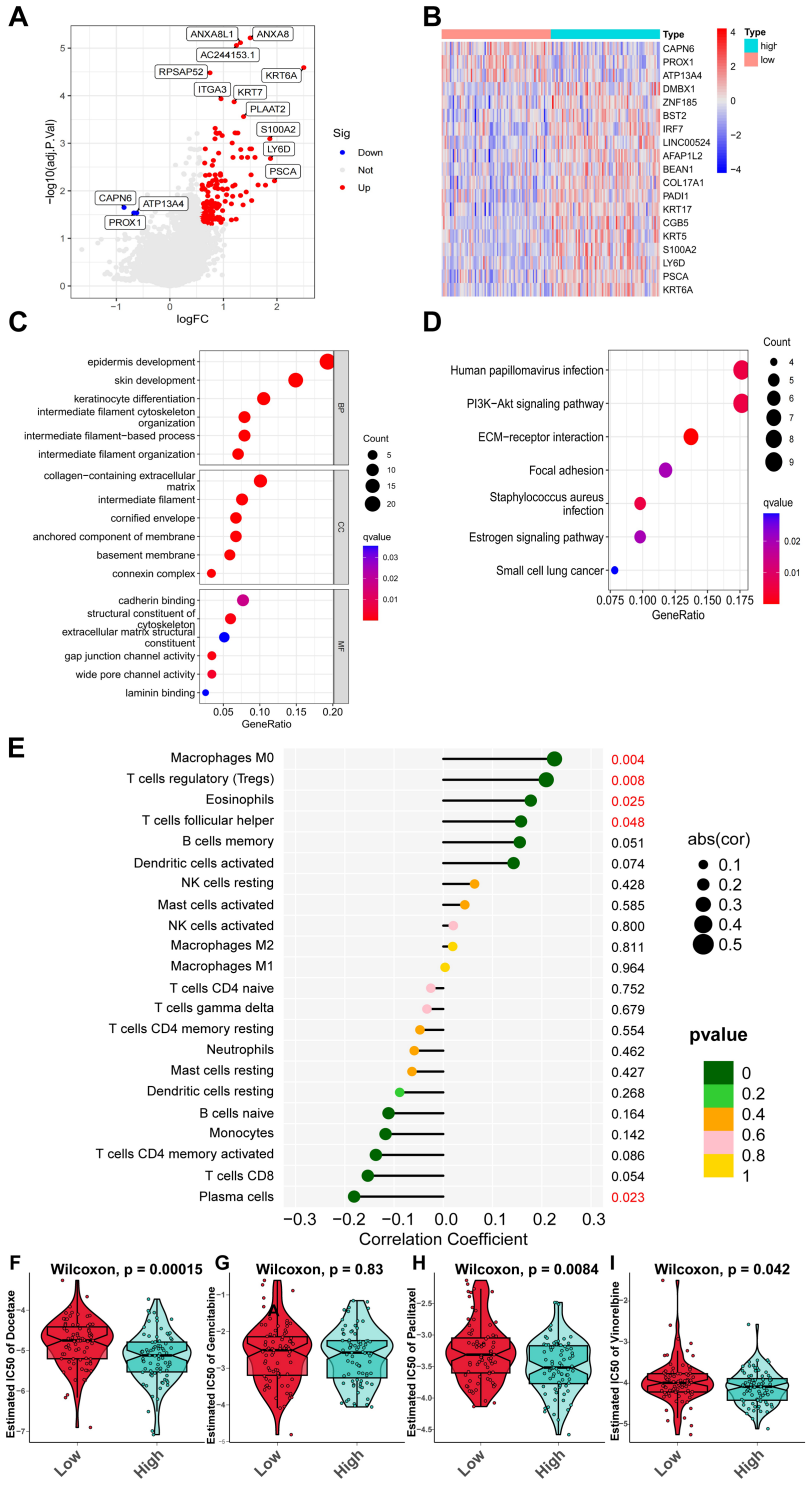
Biological functions of IL20RB and relationship with IL20RB mRNA levels and immune cells in TME. (A) The heatmap illustrated the DEGs between groups exhibiting high and low expression of IL20RB. (B) The volcano plot depicted the DEGs identified between groups with high *versus* low expression of IL20RB. (C) The bubble plot presented the results of GO enrichment analysis comparing high-expression and low-expression groups for IL20RB. (D) The bubble plot displayed the KEGG pathways associated with high-expression *versus* low-expression groups for IL20RB. (E) This panel showed the correlation between IL20RB expression levels and immune cell infiltration within the TME, where correlation coefficients were represented on the *x*-axis, while *p*-values were indicated on the *y*-axis. (F–I) A comparison of IC50 values for docetaxel, gemcitabine, paclitaxel, and vinorelbine was presented between groups with either high or low expression of IL20RB in the TCGA-PAAD cohort. GO, gene ontology; KEGG, kyoto encyclopedia of genes and genomes.

The TCGA-PAAD cohort was stratified into high-expression and low-expression groups of IL20RB, utilizing the median value of IL20RB mRNA levels as a cutoff. The DEGs between these two groups were visualized through heatmap and volcano plot representations (Figs. 10A, 10B). Subsequently, GO and KEGG pathway enrichment analyses were performed (Figs. 10C, 10D). KEGG analysis revealed several significantly enriched pathways in the high-expression group, including interactions between the extracellular matrix and receptors, the PI3K-Akt signaling pathway, focal adhesion pathway, estrogen signaling pathway, and small cell lung cancer pathway. Additionally, we analyzed the correlation between IL20RB mRNA levels and immune cell infiltration within the TME in the TCGA-PAAD cohort. The results demonstrated a positive correlation between IL20RB expression levels and macrophages (M0), regulatory T cells, eosinophils, as well as follicular helper T cells; conversely, a negative correlation was observed with plasma cells and CD8+ T cells (Fig. 10E). Furthermore, patients exhibiting elevated expression of IL20RB might show increased sensitivity to various chemotherapeutic agents such as docetaxel, gemcitabine, paclitaxel, and vinorelbine. Consequently, these patients were likely to derive enhanced therapeutic benefits from such treatments (Figs. 10F–10I). These findings were consistent with those related to TME characteristics and chemotherapy sensitivity previously identified in the CS1 subtype analysis.

## Discussion

This study identified two molecular subtypes of PDAC, namely the CS1 subtype (characterized as high-risk and immunosilent) and the CS2 subtype (defined as low-risk and immunoactive), based on multi-omics data. The CS1 subtype is characterized by elevated genomic instability, with a predominant enrichment in tumor-related pathways while exhibiting lower enrichment in immune-related pathways. Consequently, the CS1 subtype is classified as a high-risk and immunosilent variant. In contrast, the CS2 subtype demonstrateed greater genomic stability along with significant enrichment in immune-related pathways. This subtype also exhibited active immune functions, evidenced by high infiltration levels of CD8+ T cells and B cells, correlating with favorable clinical outcomes. Thus, the CS2 subtype was categorized as a low-risk and immunoactive variant. Despite the poorer clinical prognosis associated with the CS1 subtype and its reduced enrichment of immune-related pathways compared to the CS2 subtype, patients classified under the CS1 category exhibited enhanced responsiveness to chemotherapeutic agents such as paclitaxel, docetaxel, vinorelbine, and gemcitabine when compared to those within the CS2 subgroup.

This study indicates that patients with the CS1 subtype, despite a more aggressive disease course, exhibit greater intrinsic sensitivity to certain chemotherapeutic agents *in vitro*. This suggests they may derive greater therapeutic benefit from these agents than CS2 subtype patients. Nevertheless, this *in vitro* sensitivity does not translate to a less aggressive clinical course *in vivo*, as overall prognosis is governed by a complex interplay of factors including metastatic potential, rapid recurrence, and an immunosuppressive TME, all of which are hallmarks of the CS1 subtype. The intrinsic chemosensitivity of CS1 tumors may thus be counterbalanced by its aggressive features. Therefore, while CS1 patients might benefit from these chemotherapeutic agents, the findings should be interpreted cautiously. Notably, our results underscore the value of molecular multi-omics classification in optimizing first-line treatment strategies for PDAC. Additionally, the CS2 subtype, which is associated with a more favorable prognosis, exhibited significant enrichment in inflammation-related processes—specifically the IL-6/JAK/STAT3 signaling pathway and xenograft rejection mechanisms. Given the dual nature of inflammation as a vital defense mechanism that can promote pathology when dysregulated, the activation of this pathway in CS2 tumors likely signifies a robust and coordinated anti-tumor immune response—a compelling avenue for future research.

Previous studies have indicated that approximately 90% of pancreatic cancer patients harbor KRAS mutations, which are recognized as the primary driver genes in the progression of pancreatic cancer. Additionally, inactivating mutations in TP53, CDKN2A, and SMAD4 are observed in 50–80% of pancreatic cancer cases (Park, Chawla & O’Reilly, 2021). The findings from this study corroborate previous research demonstrating that the major mutated genes in PDAC include KRAS, TP53, and CDKN2A. Mutations in TP53 enhance cell proliferation processes mediated by CDK4, resulting in a poorer prognosis for younger patients diagnosed with pancreatic cancer (Tong et al., 2022). Furthermore, tumors exhibiting TP53 mutations tend to present higher TMB and tumor aneuploidy level, both of which are prevalent characteristics across various human cancers and are considered significant drivers of tumorigenesis (Li, Li & Wang, 2020). Blaveri et al. (2005) identified a correlation between FGA and poor prognosis in bladder cancer. This finding aligns with our results indicating that the CS1 subtype exhibited greater genomic instability along with increased mutation rates for KRAS, TP53, CDKN2A, PCDH15, and FLG; higher FGA levels; and a worse clinical prognosis compared to the CS2 subtype.

The TME of PDAC exhibits immunosuppressive characteristics, characterized by a predominance of inhibitory cells, a deficiency of anti-tumor immune cells, and overall immune dysfunction. This is primarily attributed to the depletion of cytotoxic T lymphocytes and the infiltration of suppressive immune cells, particularly macrophages (Bear, Vonderheide & O’Hara, 2020). These macrophages can undergo M2 polarization, which results in an increased infiltration level of regulatory T cells (Tregs) and a reduction in CD8+ T cell populations, ultimately compromising the anti-tumor efficacy of immune cells within tumor tissues (Iglesias-Escudero, Arias-Gonzalez & Martinez-Caceres, 2023). Moreover, monocytes/macrophages present in the TME are capable of secreting various pro-inflammatory factors such as TNF-*α*, IL-1*β*, and IL-6 that promote tumor growth and invasion. Additionally, these monocytes/macrophages have the potential to differentiate into tumor-associated macrophages (TAMs), which facilitate angiogenesis, invasion, and metastasis in pancreatic cancer; thus linking macrophage infiltration with poor prognostic outcomes (Yang et al., 2021). The findings from this study indicated that patients classified under the CS1 subtype exhibited elevated levels of TAMs alongside reduced levels of cytotoxic T lymphocytes resulting in poorer prognoses. In contrast, patients categorized under the CS2 subtype demonstrated higher infiltration levels of CD8+ T cells and B cells while exhibiting lower macrophage infiltration; consequently leading to improved prognoses. These observations suggested that our PDAC classification system effectively distinguished between these two subtypes for prognostic assessment while also enhancing our understanding of immune function within tumors.

The high-risk group identified in the prognostic model demonstrated lower immune and mesenchymal scores, alongside higher tumor purity. It has been proposed that “hot” or inflammatory tumors possess greater immunogenicity and are more likely to respond favorably to immunotherapy due to a heightened infiltration of immune cells. Consequently, the poor prognosis observed in patients within the high-risk group may be attributed to immunosuppression present in the TME of PDAC. GSEA further indicated that both the CS1 subtype and the high-risk group were predominantly enriched in tumor-associated pathways, whereas the CS2 subtype and low-risk group exhibited significant enrichment in immune-related pathways such as chemokine signaling, intestinal immune network, and IgA production. Therefore, it is plausible that individuals within the high-risk group may demonstrate increased activity in biological processes linked to tumor development, ultimately resulting in a poorer prognosis for patients belonging to this subgroup.

The IL20RB protein serves as a critical receptor subunit within the interleukin family, forming a heterodimeric cytokine receptor in conjunction with either IL20RA or IL22RA1. It specifically binds to cytokines from the IL-20 subfamily, which includes IL-19, IL-20, and IL-24 (Blumberg et al., 2001). Numerous studies have demonstrated that the expression of IL20RB is upregulated across various cancer types. Notably, it exhibits high expression levels in papillary renal cell carcinoma and has been shown to promote cellular proliferation, invasion, migration while correlating with poor prognosis (Cui, Cui & Leng, 2019). Furthermore, IL-20RB enhances the tumor response of osteoclastic environments to lung cancer and facilitates bone metastasis associated with this malignancy (He et al., 2022). The findings of this study reveal significantly elevated expression levels of IL20RB in PDAC, which are linked to unfavorable prognostic outcomes based on both TCGA data and clinical tissue samples. Consequently, abnormal expression of IL20RB in PDAC may serve as a potential predictor for patient prognosis. Furthermore, the mRNA levels of IL20RB were found to be positively correlated with the infiltration levels of macrophages (M0), Tregs, eosinophils, and follicular helper T cells, while exhibiting a negative correlation with CD8+ T cells. This observation is consistent with the molecular classification of PDAC and aligns with characteristics associated with high-risk or low-risk groups as defined by the PDAC prognostic model. IL20RB is expressed in a diverse array of normal cell types, including keratinocytes, fibroblasts, monocytes, T cells, and endothelial cells (Rutz, Wang & Ouyang, 2014). These findings were corroborated by analyses of single-cell RNA sequencing data that also demonstrate IL20RB expression in fibroblasts, monocytes, and endothelial cells. Pathway enrichment analysis indicated that functions and pathways related to IL20RB were primarily enriched in extracellular matrix interactions and receptor signaling, PI3K-Akt signaling pathway activation, focal adhesion processes, as well as small-cell lung cancer pathways. This suggests that IL20RB might play a more active role in biological processes associated with tumor progression, potentially leading to poorer clinical outcomes for these patients.

This study also presented several limitations. The molecular classification and prognostic models developed herein were derived from bioinformatics analyses based on online data. However, utilizing clinical data might yield more effective prognostic models. Furthermore, the translation of multi-omics techniques from laboratory settings to standard clinical applications necessitated considerable time investment and further in-depth research. A significant challenge lies in the varying maturity levels of different methodologies employed for routine clinical use (Menyhart & Gyorffy, 2021). Consequently, future efforts should focus on integrating these findings related to mRNA expression with multiplex IHC techniques to investigate the intrinsic alterations within tumor cells and their interactions with the TME, which ultimately influence treatment responses. Furthermore, this study is limited by its sample size for both data analysis and experimental validation, which may affect the generalizability of the findings. Moreover, functional validation of the identified pathway enrichments—particularly IL-6/JAK/STAT3—is crucial. Techniques such as immunohistochemistry for detecting phosphorylated proteins could provide definitive evidence of their activation within the specific molecular subtypes.

## Conclusions

In summary, our study has successfully identified two distinct molecular subtypes of PDAC through comprehensive bioinformatics analysis, each associated with unique clinical outcomes. The prognostic model developed herein demonstrates robust predictive capabilities. Furthermore, the elevated expression of IL20RB, which correlates with poor prognosis in PDAC patients, provides a novel perspective for understanding the heterogeneity of this disease and for formulating targeted treatment strategies.

##  Supplemental Information

10.7717/peerj.20619/supp-1 Supplemental Information 1 Clinicopathological characteristics of the two PDAC subtypes

10.7717/peerj.20619/supp-2 Supplemental Information 2 MIQE guidelines for real time PCR

10.7717/peerj.20619/supp-3 Supplemental Information 3 IL-20RB western blot

## Abbreviations

AUC: area under curve
CNA: copy number alteration
CPI: clustering prediction index
DEGs: differentially expressed genes
DFI: disease-free interval
DSS: disease-specific survival
ESTIMATE: estimation of stromal and immune cells in malignant tumor tissues using expression data
FGA: fraction genome altered
FGL: fraction genome loss
FGG: fraction genome gain
GEO: gene expression omnibus
GO: gene ontology
GSVA: gene set variation analysis
H&E: Hematoxylin & Eosin
IC: immune checkpoint
IC_50_: half maximal inhibitory concentration
ICIs: immune checkpoint inhibitors
IHC: immunohistochemistry
IL20RB: anti-interleukin-20 receptor subunit beta
lncRNA: long non-coding RNA
KEGG: Kyoto Encyclopedia of Genes and Genomes
Lasso: the least absolute shrinkage and selection operator
NTP: Nearest template prediction
OS: overall survival
PAAD: pancreatic adenocarcinoma
PCA: principal component analysis
PDAC: pancreatic ductal adenocarcinoma
PFI: progression-free interval
RT-PCR: real-time fluorescent quantitative PCR
ROC: receiver operation curve
ssGSEA: single-sample gene set enrichment analysis
TAMs: tumor-associated macrophages
TCGA: The Cancer Genome Atlas
TISCH2: tumor immune single-cell hub 2
TMB: tumor mutation burden
TME: tumor microenvironment
Tregs: regulatory T cells
WB: Western Blot

## Additional Information and Declarations

### Data Availability

The following information was supplied regarding data availability: The RNA-seq dataset, which includes 158 samples from patients with PAAD, is available in the TCGA database, DOI: 10.57760/sciencedb.33309. The corresponding clinical data is available at UCSC Xena, DOI: 10.57760/sciencedb.33310. The RNA-seq datasets are available at GEO: GSE183795.
