# Supplementary material for: Molecular classification and prognosis study of pancreatic ductal adenocarcinoma through multi-omics integrated clustering analysis
Source: PeerJ. 2026 Feb 16;14:e20619. doi: 10.7717/peerj.20619 (PMC12919317; doi:10.7717/peerj.20619)
Supplement: Supplemental Information 1 [file peerj-14-20619-s001.docx]

**Supplementary materials**

**Table S1 Clinicopathological characteristics of the two PDAC subtypes**

|  | Overall | CS1 subtype | CS2 subtype | *P* value |
| --- | --- | --- | --- | --- |
| Case, n (%) | 158 (100) | 76 (48.1) | 82 (51.9) |  |
| Age (year) | 64.64 ± 11.01 | 63.99 ± 11.65 | 65.24 ± 10.41 | 0.477 |
| Gender, n (%) |  |  |  | 0.966 |
| Female | 72 (45.6) | 34 (44.7) | 38 (46.3) |  |
| Male | 86 (54.4) | 42 (55.3) | 44 (53.7) |  |
| Grade, n (%) |  |  |  | 0.035* |
| G1 | 18 (11.5) | 4 (5.3) | 14 (17.5) |  |
| G2 | 91 (58.3) | 49 (64.5) | 42 (52.5) |  |
| G3 | 45 (28.8) | 23 (30.3) | 22 (27.5) |  |
| G4 | 2 (1.3) | 0 (0.0) | 2 (2.5) |  |
| T stage, n (%) |  |  |  | 0.973 |
| T1 | 5 (3.2) | 3 (3.9) | 2 (2.5) |  |
| T2 | 15 (9.6) | 7 (9.2) | 8 (9.9) |  |
| T3 | 134 (85.4) | 65 (85.5) | 69 (85.2) |  |
| T4 | 3 (1.9) | 1 (1.3) | 2 (2.5) |  |
| N stage, n (%) |  |  |  | 1 |
| N0 | 41 (26.3) | 20 (26.7) | 21 (25.9) |  |
| N1 | 115 (73.7) | 55 (73.3) | 60 (74.1) |  |
| Stage, n (%) |  |  |  | 0.689 |
| Stage 1 | 12 (7.7) | 5 (6.7) | 7 (8.6) |  |
| Stage 2 | 137 (87.8) | 66 (88.0) | 71 (87.7) |  |
| Stage 3 | 3 (1.9) | 1 (1.3) | 2 (2.5) |  |
| Stage 4 | 4 (2.6) | 3 (4.0) | 1 (1.2) |  |

Note：* indicated a significant difference (*P* < 0.05) in comparison between CS1 and CS2 subtypes.

**Table S2 Comparation analysis of the** **mutation frequency of the top 20 genes with the highest mutation frequency in the TCGA-PAAD cohort across different PDAC subtypes**

| Genes | Overall  n (%) | CS1 subtype  n (%) | CS2 subtype  n (%) | *P* value |
| --- | --- | --- | --- | --- |
| KRAS | 107 (68%) | 71 (93.4%) | 36 (43.9%) | 5.38e-12* |
| TP53 | 102 (65%) | 61 (80.3%) | 41 (50.0%) | 1.10e-048* |
| SMAD4 | 34 (22%) | 19 (25.0%) | 15 (18.3%) | 3.37e-01 |
| CDKN2A | 33 (21%) | 22 (28.9%) | 11 (13.4%) | 1.92e-02* |
| TTN | 27 (17%) | 14 (18.4%) | 13 (15.9%) | 6.79e-01 |
| MUC16 | 13 ( 8%) | 9 (11.8%) | 4 ( 4.9%) | 1.49e-01 |
| RNF43 | 11 ( 7%) | 6 (7.9%) | 5 (6.1%) | 7.59e-01 |
| CSMD2 | 8 ( 5%) | 4 (5.3%) | 4 (4.9%) | 1.00e+00 |
| RYR1 | 8 ( 5%) | 2 (2.6%) | 6 (7.3%) | 2.79e-01 |
| PCDH15 | 8 ( 5%) | 7 (9.2%) | 1 (1.2%) | 2.91e-02* |
| ARID1A | 7 ( 4%) | 3 (3.9%) | 4 (4.9%) | 1.00e+00 |
| GNAS | 9 ( 6%) | 4 (5.3%) | 5 (6.1%) | 1.00e+00 |
| OBSCN | 7 ( 4%) | 2 (2.6%) | 5 (6.1%) | 4.45e-01 |
| FLG | 8 ( 5%) | 8 (10.5%) | 0 ( 0.0%) | 2.34e-03* |
| MYO18B | 8 ( 5%) | 5 (6.6%) | 3 (3.7%) | 4.83e-01 |
| ATM | 6 ( 4%) | 2 (2.6%) | 4 (4.9%) | 6.83e-01 |
| CACNA1B | 7 ( 4%) | 5 (6.6%) | 2 (2.4%) | 2.62e-01 |
| HECW2 | 8 ( 5%) | 5 (6.6%) | 3 (3.7%) | 4.83e-01 |
| RNF213 | 7 ( 4%) | 2 (2.6%) | 5 (6.1%) | 4.45e-01 |
| TGFBR2 | 8 ( 5%) | 6 (7.9%) | 2 (2.4%) | 1.55e-01 |

Note：* indicated a significant difference (*P* < 0.05) in comparison of gene mutation frequency between CS1 and CS2 subtypes.

**Table S3 The top 100 genes specifically upregulated in each PDAC subtype**

| CS1 subtype | | |  | CS2 subtype | | |
| --- | --- | --- | --- | --- | --- | --- |
| CASP14 | KRT16 | SLURP2 |  | DEFA5 | AC098935.2 | CMA1 |
| MAGEA3 | AC112482.1 | LIPK |  | DEFA6 | CELA3A | AL139020.1 |
| FGF23 | MYO16-AS1 | MIR210 |  | PNLIP | RBPJL | VPREB3 |
| SLURP1 | MIR205HG | RPTN |  | CLPS | SPX | TRARG1 |
| MUC21 | IGFL2-AS1 | LINC02742 |  | CSN1S1 | CD19 | PCDH15 |
| UPK2 | LINC01776 | AC027128.1 |  | LINCADL | CPA1 | PLIN1 |
| PAX7 | Z94160.1 | LINC01219 |  | THRSP | NIBAN3 | GP2 |
| AC034223.1 | FAHD2P1 | AC018978.1 |  | TCL1A | AF131216.3 | PLA2G1B |
| LINC01929 | NTF4 | AC025252.2 |  | FCRL1 | AMY2B | AC022239.1 |
| AC034223.2 | AC023421.2 | SLC14A1 |  | SCGB2A2 | BPIFB4 | KRT73 |
| CST8 | AC021713.1 | AC090505.2 |  | SMYD1 | AQP12B | CELP |
| KRT6A | MROH9 | AC124276.1 |  | REG3A | MYOC | LINC01781 |
| AC023824.3 | CYMP-AS1 | HAVCR1 |  | AL161781.2 | CEL | PNLIPRP1 |
| AC083841.2 | IL36RN | IVL |  | AMY2A | COL19A1 | PTF1A |
| LINC01940 | AL354766.2 | AC005753.2 |  | RN7SL386P | FGL1 | AC002546.1 |
| CGB5 | NKX2-8 | AC007950.1 |  | LINC00469 | MS4A1 | MIR4539 |
| KRT4 | AC083841.1 | AL139420.1 |  | AGTR2 | CIDEA | NLRP4 |
| LINC01322 | SERPINB3 | ICAM5 |  | AC133065.2 | SCARA5 | TBC1D27P |
| CA9 | SPINK7 | LINC02448 |  | PLA2G2A | AL121835.2 | SERPINI2 |
| AC107308.1 | EPS15P1 | AC079070.1 |  | SYCN | U62631.1 | BANF2 |
| AC104453.1 | KRT9 | AL590666.4 |  | CD300LG | LINC02397 | ITLN1 |
| AC010343.3 | GUCY2EP | AL031848.1 |  | FCER2 | REG1CP | RETNLB |
| LINC01077 | FAM83A | RNF225 |  | FREM2-AS1 | PRAMENP | AC108879.1 |
| LINC02300 | LINC02122 | LINC00431 |  | GUCA1C | TMED11P | PPY |
| TNNT1 | EREG | H2AC9P |  | GPHA2 | BPIFB2 | LINC00402 |
| LINC02411 | AL161431.1 | HIF1A-AS3 |  | LEP | FAM167A-AS1 | CPB1 |
| CYMP | MYH16 | AC008687.3 |  | ADIPOQ | BLK | FCRLA |
| LY6D | LINC00462 | AC011601.1 |  | CELA2A | AL590556.3 | AMYP1 |
| LINC02178 | IL36B | AC091173.1 |  | LINC02305 | SLC7A10 | CELA2B |
| IL20RB | KLHDC7B | LYPD2 |  | CPA2 | CELA3B | PAX5 |
| AL109615.2 | ARL14EPL | AC064856.1 |  | MIR4538 | CNR2 | AQP8 |
| S100A2 | UCA1 | H2BC16P |  | REG3G | AQP12A | SCGB3A1 |
| A2ML1 | EGLN3-AS1 | AC078816.1 |  | LINC01811 | MIR4537 | AL109910.2 |
| HSPA6 |  |  |  | PRSS1 |  |  |

**Table S4 Relationship between IL20RB protein and clinical pathological characteristics of PDAC**

| Parameters | | Overall | IL20RB Positive | IL20RB Negative | *P* value |
| --- | --- | --- | --- | --- | --- |
| Age (year) | | 61.99 ± 10.12 | 61.62 ± 10.03 | 62.15 ± 10.26 | 0.843 |
| Gender, n (%) | | | |  | 1 |
|  | Male | 43 (63.2) | 13 (61.9) | 30 (63.8) |  |
|  | Female | 25 (36.8) | 8 (38.1) | 17 (36.2) |  |
| Size of PDAC (cm) | | 4.13 ± 1.85 | 4.07 ± 1.53 | 4.16 ± 1.99 | 0.838 |
| pT stage, n (%) | | | | | 0.604 |
|  | T1 | 7 (10.3) | 1 (4.8) | 6 (12.8) |  |
|  | T2 | 33 (48.5) | 10 (47.6) | 23 (48.9) |  |
|  | T3 | 28 (41.2) | 10 (47.6) | 18 (38.3) |  |
| pN stage, n (%) | | | | | 0.729 |
|  | N0 | 31 (45.6) | 11 (52.4) | 20 (42.6) |  |
|  | N1 | 27 (39.7) | 8 (38.1) | 19 (40.4) |  |
|  | N2 | 10 (14.7) | 2 (9.5) | 8 (17.0) |  |
| pM stage, n (%) | | | | | 0.641 |
|  | M0 | 63 (92.6) | 19 (90.5) | 44 (93.6) |  |
|  | M1 | 5 (7.4) | 2 (9.5) | 3 (6.4) |  |
| Nerve invasion, n(%) | | | |  | 0.658 |
|  | Absence | 6 (8.8) | 1 (4.8) | 5 10.6) |  |
|  | Presence | 62 (91.2) | 20 (95.2) | 42 (89.4) |  |
| Vessel invasion, n(%) | | | |  | \| 0.361 \| \| --- \| |
|  | Absence | 53 (77.9) | 18 (85.7) | 35 (74.5) |  |
|  | Presence | 15 (22.1) | 3 (14.3) | 12 (25.5) |  |
| Histological grade, n (%) | | | |  | 0.46 |
|  | Well | 8 (11.8) | 4 (19.0) | 4 (8.5) |  |
|  | Moderate | 37 (54.4) | 10 (47.6) | 27 (57.4) |  |
|  | Poor | 23 (33.8) | 7 (33.3) | 16 (34.0) |  |

| 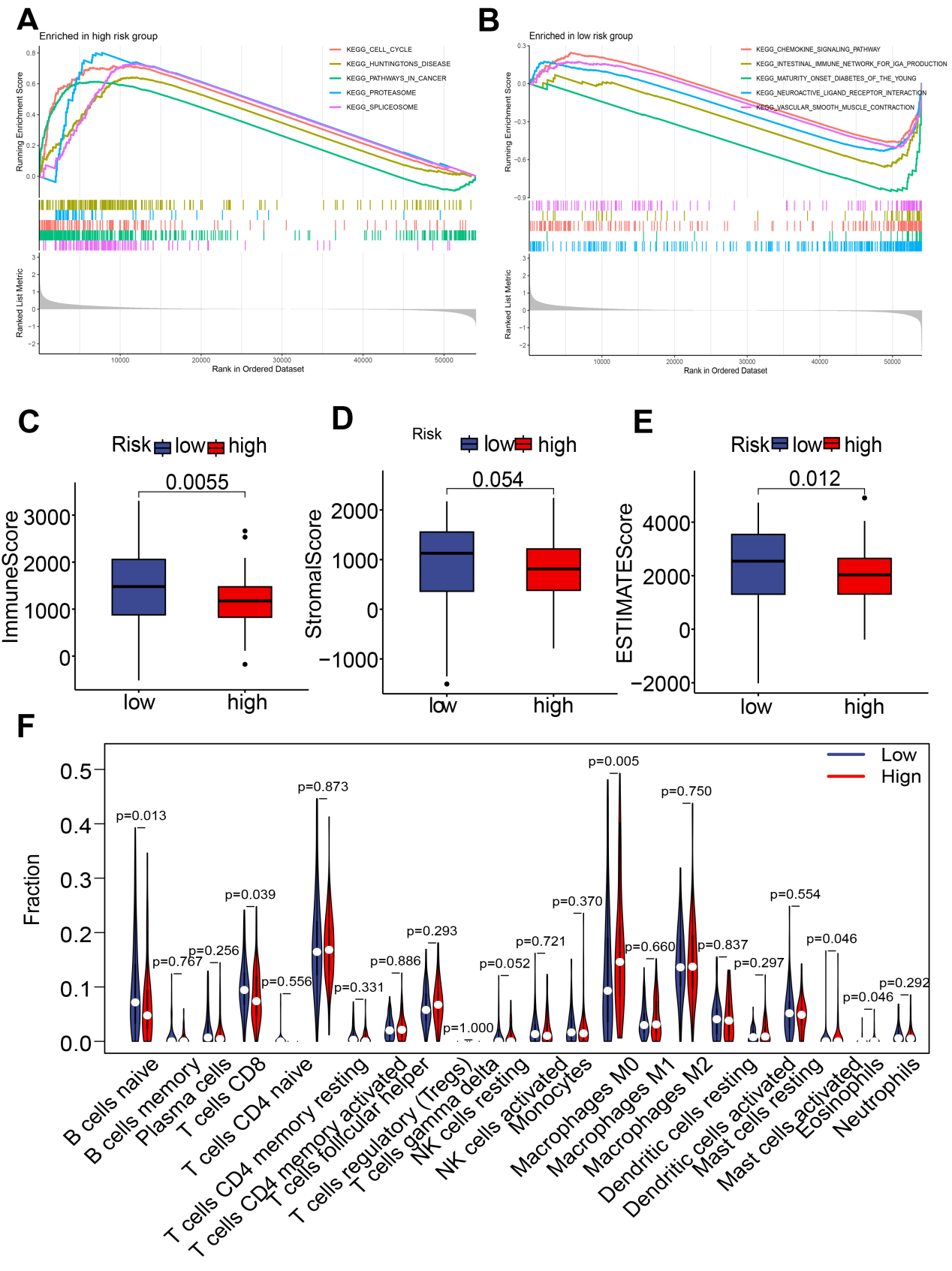 |
| --- |
| **Fig. S1 Assessment of enriched pathways and TME analysis in high-risk and low-risk groups of the TCGA-PAAD cohort.** (A) Pathways that were significantly enriched in the high-risk group, as determined by GSEA. (B) Pathways that were significantly enriched in the low-risk group, as identified through GSEA. (C-E) A comparative analysis of stromal score, immune score, and ESTIMATE score between the high-risk and low-risk groups revealed elevated scores across all three assessments for the low-risk cohort. (F) Variations in TME immune cell populations between the high-risk and low-risk groups. GSEA: gene set enrichment analysis; TME: tumor microenvironment. |
