# Supplementary material for: Molecular classification and prognosis study of pancreatic ductal adenocarcinoma through multi-omics integrated clustering analysis
Source: PeerJ. 2026 Feb 16;14:e20619. doi: 10.7717/peerj.20619 (PMC12919317; doi:10.7717/peerj.20619)
Supplement: Supplemental Information 2 [file peerj-14-20619-s002.docx]

|  | MIQE Guidelines for Real-Time PCR:  Checklist for Authors, Reviewers, and Editors | | |  |
| --- | --- | --- | --- | --- |
| Alessential MIQE information (E) must be submitted with the manuscript (see Bustin et al. 2009). Desirable information (D) should be submitted if available. If primers were obtained from RTPrimerDB, information on qPCR target, oligonucleotides, protocols, and validation is available from that source. | | | | |
| Item | | MIQE | Comments/My Information | Page numbers in the manuscript |
| Experimental Design | | | |  |
| Definition of experimental and control groups | | E | Pancreatic ductal adenocarcinoma (PDAC) and adjacent non-tumor tissues. | 12 |
| Number within each group | | E | 6 pairs (PDAC and matched adjacent tissues) | 12 |
| Assay location; core or investigator's laboratory | | D |  |  |
| Acknowledgement of authors'contributions | | D |  |  |
| Samples | | | |  |
| Description | | E | Six cases of PDAC tissues and six cases of adjacent normal tissues. | 12 |
| Volume/mass of sample processed | | D |  |  |
| Microdissection or macrodissection | | E | Macrodissection | 12 |
| Processing procedure | | E | The collected tissues were immediately snap-frozen in liquid nitrogen. |  |
| If frozen -how and how quickly? | | E | The collected tissues were immediately snap-frozen in liquid nitrogen. |  |
| If fixed-with what, how quickly? | | E | The collected tissues were immediately snap-frozen in liquid nitrogen without fixation. |  |
| Sample storage conditions and duration  (especially for FFPE samples) | | E | The tissue samples were stored in liquid nitrogen, and experiments were initiated after two month of storage. |  |
| Nucleic Acid Extraction | | | |  |
| Procedure and/or instrumentation | | E | Total RNA was extracted using a spin column-based method. | 12 |
| Kit name; details of any modifications | | E | RNeasy Mini Kit, Qiagen, USA | 12 |
| Source of additional reagents used | | D |  |  |
| Details of DNase or RNase treatment | | E | To minimize potential RNA degradation by environmental RNases, strict RNase-free techniques were implemented throughout all procedures:  Personal Protection: Wore disposable nitrile gloves; Used sterile hair caps.  Work Area Preparation: Disinfected all work surfaces with 70% ethanol before experimentation.  RNase-Free Materials: Exclusively used certified RNase-free consumables: Aerosol-resistant filter pipette tips, DNase/RNase-free microcentrifuge tubes, Pre-sterilized plasticware.  Temperature Control: Maintained all samples and reagents on ice during handling. Performed critical steps in a 4℃ cold room when necessary. |  |
| Contamination assessment (DNA or RNA) | | E | RNA concentration and purity were spectrophotometrically determined by measuring absorbance at 260 nm (A260) and calculating the following ratios: A260/A280-Protein contamination indicator, A260/A230-Organic compound/salt contamination indicator. Ensure that the A260/A280 ratio is maintained within the range of 1.8 to 2.0, and that the A260/A230 ratio falls within the range of 2.0 to 2.2. |  |
| Nucleic acid quantification | | E | RNA concentration was measured spectrophotometrically, and the total RNA mass was calculated by: Total RNA Mass (μg) = RNA Concentration (μg/μL) × Sample Volume (μL). |  |
| Instrument and method | | E | RNA concentration was measured spectrophotometrically. |  |
| Purity (A_260_/A_280_) | | D |  |  |
| Yield | | D |  |  |
| RNA integrity method/instrument | | E | RIN≤8; Agilent 2100 Bioanalyzer |  |
| RIN/RQl or Cq of 3'and 5'transcripts | | E | RIN=8.1 |  |
| Electrophoresis traces | | D |  |  |
| Inhibition testing (Cq dilutions, spike, or other) | | E | To assess PCR inhibition, cDNA samples were serially diluted (1:5, 1:25) and analyzed by qPCR. The ΔCq between dilutions was 2.32, indicating no significant inhibition. |  |
| Reverse Transcription | | | |  |
| Complete reaction conditions | | E | First-Strand cDNA Synthesis: 37°C for 15 minutes (optimal activity of reverse transcriptase) ,  Enzyme Inactivation: 85°C for 5 seconds (heat denaturation to terminate reaction) ,  Reaction Termination: 4°C hold indefinitely (immediate cooling to preserve cDNA stability). |  |
| Amount of RNA and reaction volume | | E | 500ng;10ul |  |
| Priming oligo (if using GSP) and concentration | | E | 50 uM, 0.5ul |  |
| Reverse transcriptase and concentration | | E | PrimeScript RT Enzyme Mix, 0.5ul |  |
| Temperature and times | | E | First-Strand cDNA Synthesis: 37°C for 15 minutes,  Enzyme Inactivation: 85°C for 5 seconds,  Reaction Termination: 4°C hold indefinitely. |  |
| Manufacturer of reagents and product codes | | D |  |  |
| Cqs with and without RT | | D |  |  |
| Storage conditions of cDNA | | D* | liquid nitrogen |  |
| qPCR Target Information | | | |  |
| Gene symbol | | E | IL20RB |  |
| Sequence accession number | | E | 53833 |  |
| Location of amplicon | | D |  |  |
| Amplicon length | | E | 158bp |  |
| In silico specificity screen (BLAST,etc.) | | E | Primer specificity was verified by:  1. NCBI Primer-BLAST (GRCh38.p13) with default parameters (word_size=7, E-value threshold=0.1), confirming no off-target hits.  2. All primers target exon-exon junctions (forward: exon 3; reverse: exon 4) to avoid genomic DNA amplification.  3. No pseudogenes or homologs were identified via UCSC In-Silico PCR. |  |
| Pseudogenes,retropseudogenes,  or other homologs? | | D |  |  |
| Sequence alignment | | D |  |  |
| Secondary structure analysis of amplicon | | D |  |  |
| Location of each primer by exon or intron  (if applicable) | | E | - Forward primer: 3'-end of exon 3 (NM_144727.3)  - Reverse primer: 5'-end of exon 4 (NM_144727.3)  The 158 bp amplicon spans the exon 3-4 junction, ensuring specific detection of spliced IL20RB mRNA while excluding genomic DNA (gDNA) amplification. |  |
| What splice variants are targeted? | | E | The primers (spanning exon 3-exon 4 junction) are designed to specifically amplify IL20RB splice variants that retain both exons, including: Canonical transcript (NM_144727.3) Variant 1 (NM_001363735.1) Variant 2 (NM_001363736.1). |  |
| qPCR Oligonucleotides | | | |  |
| Primer sequences | | E | The primer sequences for human IL-20RB were as follows: forward primer sequence 5'-AGGCCCAGACATTCGTGAAG-3', and reverse primer sequence 5'-GCATGAAGCCAACAAAGGCA-3'. | 13 |
| RTPrimerDB identification number | | D |  |  |
| Probe sequences | | D** |  |  |
| Location and identity of any modifications | | E | The primers were unmodified. No probes were used in this SYBR Green-based assay. |  |
| Manufacturer of oligonucleotides | | D |  |  |
| Purification method | | D |  |  |
| qPCR Protocol | | | |  |
| Complete reaction conditions | | E | qPCR was run on a Real-time quantitative PCR system (Roche, Switzerland) with:  - 95℃ for 30 sec (enzyme activation)  - 40 cycles of:  - 95℃ for 5 sec  - 60℃ for 30 sec (SYBR Green signal acquisition)  - Melt curve: 65℃→95℃, 0.5℃ increments every 5 sec. |  |
| Reaction volume and amount of cDNA/DNA | | E | 20ul |  |
| Primer (probe), Mg#*, and dNTP concentrations | | E | Primers (0.4 μM each)，3 mM MgCl₂, and 0.2 mM each dNTP. |  |
| Polymerase identity and concentration | | E | TB Green® Premix Ex Taq™ II (Takara, RR820A) was used, which contains a proprietary blend of Hot Start Taq DNA Polymerase and Ex Taq HS DNA Polymerase at manufacturer-optimized concentrations. | 13 |
| Buffer/kit identity and manufacturer | | E | TB Green® Premix Ex Taq™ II (Takara Bio, RR820A) was used, which contains Hot Start Taq DNA Polymerase, dNTPs, MgCl₂ (3 mM final), and TB Green fluorescent dye in an optimized buffer system. | 13 |
| Exact chemical constitution of buffer(s) | | D |  |  |
| Additives (SYBR Green I, DMSO, etc.) | | E | SYBR Green | 13 |
| Plate/tube manufacturer product codes | | D |  |  |
| Complete thermocycling parameters | | E | qPCR was run on a Real-time quantitative PCR system (Roche, Switzerland) with:  - 95℃ for 30 sec (enzyme activation)  - 40 cycles of:  - 95℃ for 5 sec  - 60℃ for 30 sec (SYBR Green signal acquisition)  - Melt curve: 65℃→95℃, 0.5℃ increments every 5 sec. |  |
| Reaction setup (manual/robotic) | | D |  |  |
| Manufacturer of qPCR instrument | | E | a Real-time quantitative PCR system (Roche, Switzerland, LightCycler® 480) |  |
| qPCR Validation | | | |  |
| Evidence of optimization (from gradients) | | D |  |  |
| Specificity (gel, sequence, melt, or digest) | | E | Specificity was verified by:  - Single peak in melt curve analysis (Tm = 82.5 ± 0.3℃)  - No primer-dimer artifacts (no secondary peaks below 75℃) |  |
| For SYBR Green I, Cq of the NTC | | E | NTCs (n=3) showed undetectable amplification (Cq ≥ 40) |  |
| Standard curves with slope and y-intercept | | E | Standard curves were generated from 10-fold serial dilutions of purified PCR product (10^8^–10^2^ copies/μL).  - Slope: -3.35 ± 0.02  - Y-intercept: 36.8  - R²: 0.998  - Efficiency: 99% ± 1% |  |
| PCR efficiency calculated from slope | | E | Standard curves were generated from 10-fold serial dilutions of purified PCR product (10^8^–10^2^ copies/μL).  - Slope: -3.35 ± 0.02  - Y-intercept: 36.8  - R²: 0.998  - Efficiency: 99% ± 1% |  |
| Confidence interval for PCR efficiency  or standard error | | D |  |  |
| R^2^ of standard curve | | E | 0.998 |  |
| Linear dynamic range | | E | 10^8^–10^2^ copies/μL (R² = 0.998) |  |
| Cq variation at lower limit | | E | At LLOQ (10 copies/μL):  - Mean Cq = 35.2 ± 1.8 (n=6)  - CV = 5.1% |  |
| Confidence intervals throughout range | | D |  |  |
| Evidence for LOD | | E | LOD determination:  1. Tested 1-20 copies/μL (n=20 replicates each)  2. Fitted probit curve:  - 95% LOD = 3.2 copies/μL (95%CI: 2.1-5.0)  3. Verified: 3 copies/μL showed 19/20 detection. |  |
| If multiplex assay, efficiency and LOD of each assay | | E | - Gene IL20RB: Efficiency = 99% (Slope: -3.35, R² = 0.998), LOD = 3.2 copies/μL (95% CI: 2.1–5.0).  - Reference Gene β-actin: Efficiency = 98% (Slope: -3.38, R² = 0.997), LOD = 2.8 copies/μL. |  |
| Data Analysis | | | |  |
| qPCR analysis program (source, version) | | E | Roche, Switzerland |  |
| Cq method determination | | E | Threshold: Fixed (0.2), baseline: cycles 3–15 Cq  Calculation: Second Derivative Max (Roche LightCycler® 480 v1.5.1) |  |
| Outlier identification and disposition | | E | - Criteria: Cq SD > 0.5 or abnormal amplification curve  - Action: Excluded and repeated; marked if irreproducible  - Validation: Re-tested with new RNA extraction (n=3) |  |
| Results of NTCs | | E | NTCs (n=3) showed undetectable amplification (Cq ≥ 40), confirming absence of contamination. |  |
| Justification of number and choice of reference genes | | E | The reference gene (β-actin) is selected based on:  1) Stability analysis by geNorm (M-value < 0.5 in all sample groups);  2) Consistent expression across PDAC and adjacent normal tissues (CV < 5%);  3) Prior validation in pancreatic cancer studies. | 13 |
| Description of normalization method | | E | Normalization was performed using the ΔΔCq method:  1) Target gene Cq values were normalized to the geometric mean of one reference genes (β-actin), selected based on stability analysis (geNorm M-value < 0.5). M-value = 0.3.  2) Relative expression was calculated as ( 2^{-ΔΔCq}), where ΔΔCq = (Cq_target - Cq_reference)_sample - (Cq_target - Cq_reference)_calibrator.  3) No-RT controls (Cq ≥ 40) confirmed no genomic DNA interference. | 13 |
| Number and concordance of biological replicates | | D |  |  |
| Number and stage(RT or qPCR) of technical replicates | | E | RT: n=2; qPCR: n=3 |  |
| Repeatability (intra-assay variation) | | E | %CV = 1.2% (n=3) |  |
| Reproducibility (inter-assay variation, %CV) | | D |  |  |
| Power analysis | | D |  |  |
| Statistical methods for result significance | | E | For comparisons between two groups, independent samples t-test was used for normally distributed data, while the Mann-Whitney U test was applied for non-normally distributed data. | 14 |
| Software (source,version) | | E | R language (Version 4.2.0) | 14 |
| Cq or raw data submission using RDML | | D |  |  |
| *Assessing the absence of DNAusing a no-reverse transcription (no-RT assay is ssential when frst extracting RNA.Once the sample has been vafdated as RDNA-re, the inclusion of ano-RT control is desirable but no longer essential. | | | |  |
| ** Disclosureofthe probe sequence ishighy desirable and strongly encouraged. However, since notal commercial predesigned assay vendors provide this nformation, it cannot be an essential requirement.Therefore,the use of assays that do not provide context sequences is not advised. | | | |  |
| BLAST Basic Local Aigment Search To; Cq, quantifcation cycle; CV, coeffcient of varation; DMSO, dimethyl sufoxide; FPE, formalin-ixed parfn-embeded; GSP, gene-specific primer; LOD limit of detection; MIOE, minimum information for pubication of quantitatiereal-time PCR experiments; NTC, no template controt; qPCR, quantaive PCR; RDML, Real-Time PCR Data Markup Language; RDNA, residual DNA; RIN, RNA integrity umber; ROI, RNA quality indicator; RT, reverse transcription; RTPrimerDB, freely accessible database and analysis tool for real-time quantitative PCR assays. | | | |  |
| Reference | | | |  |
| Bustin SA et al.(2009).The MIQE guidelines:Minimum information for publication of quantitative real-time PCR experiments.Clin Chem 55,611-622. BIO-RADis a rademark of Bio-Rad Laboratories,Inc.Al trademarks used herein are the property of their respective owner. 2023 Bio-Rad Laboratories,Inc. SYBR is a trademark of Themo Fisher Scientific Inc. | | | |  |
